# A Blockchain-Based Hybrid ABAC–CapBAC Framework for Secure IoT Access Control

**DOI:** 10.3390/s26217072

**Published:** 2026-11-09

**Authors:** Fatimah Alghamdi, Manal Bayousef

**Affiliations:** Department of Information Technology, Faculty of Computing and Information Technology, King Abdulaziz University, Jeddah 21589, Saudi Arabia; mbayousif@kau.edu.sa

**Keywords:** security, internet of things, access control, blockchain, hyperledger fabric

## Abstract

Growing Internet of Things (IoT) deployments require access control for heterogeneous and resource-constrained devices without excessive repeated-authorization overhead. This study proposes and evaluates a blockchain-based hybrid access-control framework that combines Attribute-Based Access Control (ABAC) and Capability-Based Access Control (CapBAC) using Hyperledger Fabric. ABAC evaluates initial requests using certificate-derived user attributes and the requested device and action, while successful authorization produces a digitally signed capability token for subsequent requests. Hyperledger Fabric maintains policy and token-state records, and a Smart Gateway validates signed tokens through either direct Ledger retrieval or an event-synchronized local Cache. The framework was implemented as a proof-of-concept with simulated IoT devices on a single host and evaluated for functional correctness and repeated-validation performance. Functional tests rejected unauthorized requests, tampered, revoked, and expired tokens, and device/action mismatches. In the main repeated-validation benchmark, Cache-mode throughput was 1.50–1.87 times Ledger-mode throughput. At 1000 concurrent requests, Cache validation reduced mean request latency from 2099.45 ms to 1120.88 ms, a reduction of 46.61%, and increased mean throughput from 221.59 to 358.16 requests per second, corresponding to 1.62 times the Ledger-mode throughput. Under the evaluated prototype conditions, the framework provided attribute-based initial authorization and reusable signed-token validation, while synchronized Gateway caching improved repeated-access performance.

## 1. Introduction

**Figure 1:**
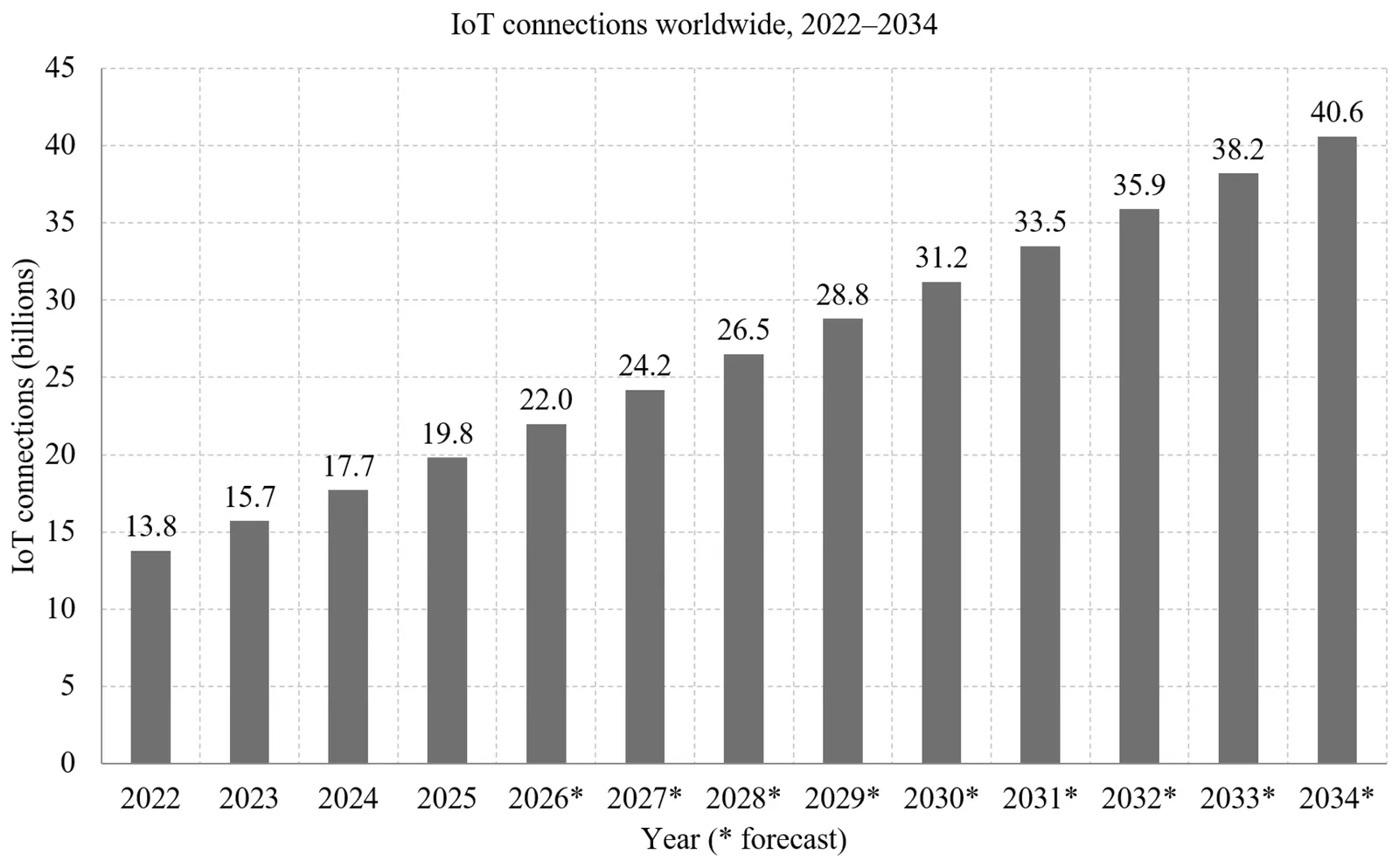
Number of Internet of Things (IoT) connections worldwide, 2022–2034 (in billions). Authors’ visualization based on data from Statista [3]. An asterisk (*) indicates forecast values.

The Internet of Things (IoT) connects billions of heterogeneous devices, sensors, gateways, and software services to support data collection, transmission, processing, and interpretation for subsequent actions [1,2]. The number of IoT connections worldwide is forecast to reach 40.6 billion by 2034, as shown in Figure 1 [3]. IoT applications span several domains, including smart homes, healthcare, industrial systems, and smart cities, where connected devices, gateways, and software services interact to collect, transmit, and process data [1,2]. As IoT environments grow, access control becomes essential for security to ensure that only authorized users, devices, and services can access protected resources and data [1,2]. Unlike traditional Information Technology environments, IoT systems are highly heterogeneous, interconnected, and often composed of resource-constrained devices, which makes security enforcement more challenging [1]. Without effective access control, IoT systems may face unauthorized access, data breaches, operational disruptions, privacy issues, and misuse of connected devices [1,2].

Traditional access control systems face limitations in IoT environments because of centralization, scalability issues, latency, and the limited resources of IoT devices. Centralized systems can create single points of failure and increase management workload in large-scale deployments. Additionally, IoT systems remain vulnerable to threats such as unauthorized access, man-in-the-middle attacks, device impersonation, and denial-of-service attacks [4,5,6].

Blockchain technology offers a promising solution with decentralization, transparency, immutability, and secure data management. It allows distribution of access control policies and decisions across multiple nodes, which reduces dependence on a central authority and supports secure and auditable operations [7]. However, integrating blockchain with IoT is challenging because IoT devices often lack processing power, storage, and energy. Therefore, lightweight access control methods and efficient storage solutions are necessary to make blockchain-based options feasible for IoT environments [4,5].

Current access control models have various advantages and limitations. Role-Based Access Control (RBAC) is straightforward and lightweight but lacks flexibility in dynamic IoT settings [8]. Attribute-Based Access Control (ABAC) enables detailed and flexible policy evaluation, as shown in [9,10,11,12,13,14,15,16,17], but may require more computational power. Capability-Based Access Control (CapBAC), discussed in [18,19,20], offers scalable and lightweight access enforcement but is less adaptable to changing policies. Hybrid models like ABAC–RBAC have also been explored [21]; however, the reported study evaluates simulated smart-contract operations and does not report transaction throughput or network latency.

This paper implements and evaluates a hybrid ABAC–CapBAC framework on Hyperledger Fabric. ABAC evaluates access requests using certificate-derived attributes such as role and department together with request attributes, including device identifier (ID) and requested action. After access approval, CapBAC creates the token state on-chain, while an off-chain Signer generates the reusable signed capability token, reducing repeated policy evaluation and improving access-enforcement efficiency.

Hyperledger Fabric was selected as the blockchain platform because of its permissioned architecture, modular and extensible design, scalability, and smart-contract capabilities [22]. These characteristics make it suitable for secure and efficient access control in IoT environments. The framework also employs an off-chain Signer to generate signed capability tokens and an event-driven Gateway Cache to maintain a synchronized, Ledger-derived copy of token states for efficient access validation.

The main contributions of this paper are as follows:

1. Implementation of a proof-of-concept that performs the initial ABAC evaluation and records the complete token state on Hyperledger Fabric, while using off-chain Ed25519 signing and Smart Gateway enforcement of reusable capability tokens.
2. Enhancement of access-control flexibility through attribute-based authorization at token issuance using the requester’s role and department, the target device, the requested action, and, when specified by the applicable policy, an access-time window.
3. Enhancement of security through secure access records and decentralized management of policies and tokens on Hyperledger Fabric, Ledger-authoritative token revocation, and digitally signed capability tokens that support verification of token integrity and authenticity.

The empirical evaluation addresses the following research questions:

RQ1: To what extent does the proposed hybrid ABAC–CapBAC framework correctly perform its intended access-control functions and reject unauthorized or invalid signed-token requests under the tested scenarios?
RQ2: How does the Gateway Cache affect repeated signed-token validation performance, in terms of batch completion time, mean request latency, and throughput, compared with Ledger-based validation under increasing concurrent workloads?

Motivated by the sensitivity of healthcare data and the need for secure access control in healthcare applications [23], this study targets a healthcare-oriented IoT environment in which connected medical devices must be accessed only by authorized users.

The remainder of this paper is organized as follows: Section 2 reviews the related work, Section 3 describes the methodology, Section 4 presents the results, Section 5 presents the discussion, and Section 6 concludes the paper.

## 2. Related Work

The rapid growth of the Internet of Things (IoT) has introduced major access control challenges, particularly in environments that require scalability, security, flexibility, and lightweight operation. Traditional centralized access control systems suffer from several limitations, including single points of failure, scalability bottlenecks, and limited adaptability to dynamic IoT environments [4,5]. Blockchain technology has been widely investigated as a promising solution because of its decentralized, tamper-resistant, transparent, and auditable nature [6].

This section reviews existing blockchain-based access control models for IoT environments. Several studies have explored role-based and hybrid access control models using blockchain.

Usman et al. [8] proposed a blockchain-based RBAC framework using Hyperledger Fabric for Industrial IoT environments. Their framework uses chaincodes to manage permissions, support delegation, and resolve policy conflicts. The evaluation used concurrent virtual clients to examine computational cost and scalability under increasing workloads. Because the evaluation was simulation-based, the reported results characterize the framework under the tested workloads; performance in a physical industrial deployment was not reported. Similarly, Noor et al. [21] proposed a hybrid ABAC–RBAC model using Ethereum for smart agriculture. Their framework combines role-based and attribute-based decisions to improve flexibility and scalability in heterogeneous IoT environments. However, the study did not provide sufficient real-world performance metrics or large-scale scalability evaluation. A large portion of the literature focuses on ABAC because it supports fine-grained and dynamic access control decisions based on attributes.

Shammar et al. [9] proposed a Hyperledger Fabric-based ABAC model using the Raft consensus mechanism to improve throughput and response time in IoT environments. Shih et al. [10] also proposed an ABAC model for Industrial IoT using multiple smart contracts, including device, policy, and access contracts. Iftekhar et al. [11] demonstrated the deployment of Hyperledger Fabric with ABAC on ARM64-based IoT devices, showing its suitability for low-power environments.

Further studies applied ABAC using smart contracts on other blockchain platforms. Zaidi et al. [12] proposed a dual-chain architecture combining Ethereum and MultiChain to improve scalability and security, while Yang et al. [14] integrated blockchain with attribute-based encryption to provide secure and traceable IoT data access. Although these studies demonstrate the benefits of ABAC in providing fine-grained access control, many of them lack large-scale real-world testing, detailed performance benchmarking, or evaluation under heavy IoT workloads.

Other works have investigated Hyperledger Fabric-based access control frameworks for IoT. Zhang et al. [13] developed a collaborative ABAC framework with emergency access support using Hyperledger Fabric chaincode. Liu et al. [15] proposed Fabric-IoT, a blockchain-based ABAC system that enforces policies through smart contracts and improves performance in distributed IoT environments. Roy and Ghosh [16] proposed BloAC, a decentralized IoT access-control framework that uses Hyperledger Fabric and customized ABAC policies. The ABAC policies are encoded in chaincode, while the Fabric network is hosted by edge and gateway nodes to provide access control without relying on a back-end cloud. The framework was evaluated through simulation and a four-Raspberry-Pi testbed, and the results showed lower latency than cloud-server-based access control. However, the authors identified initial setup overhead, scalability and interoperability challenges in large-scale deployments, and the static assignment of blockchain roles to edge nodes (e.g., committer, endorser, and orderer) as limitations. More recently, Ullah et al. [17] proposed DGAC-IoT for cross-domain IoT environments, in which ABAC policies are evaluated off-chain at domain gateways and authorization decisions are cached within explicit validity intervals. Hyperledger Fabric serves as a distributed trust anchor that records signed authorization commitments, policy updates, and revocation events. DGAC-IoT was evaluated under increasing request rates, different cache-hit ratios, and dynamic revocation conditions. In the present framework, the initial ABAC decision and the creation of the complete token state occur in chaincode under the applicable endorsement policy, while an off-chain Signer signs the token claims to produce a reusable capability token that the user presents to the Gateway. The Gateway verifies the signature and validates the token claims against the token state retrieved directly from the Ledger or from a local cache; consequently, cache hits avoid repeated Ledger retrieval. Revocation changes the token status on the Ledger, and event processing updates the cached record. Thus, the two frameworks differ primarily in the placement of ABAC evaluation, the representation of authorization state, and the handling of reusable tokens. These architectural differences do not, by themselves, establish performance superiority over DGAC-IoT. Capability-based access control has also been explored as a lightweight solution for IoT. Nakamura et al. [18] proposed an Ethereum-based CapBAC scheme that defines one capability token per authorized action and uses a delegation graph to manage delegation relationships. Smart contracts support token creation, delegation, revocation, and verification of token ownership and validity. The prototype was implemented on a private Ethereum network using two computers and two Raspberry Pi devices. The evaluation demonstrated the implemented functions and measured gas consumption for token creation, delegation, and revocation. The authors identified limited privacy of access rights because tokens are stored on the blockchain without encryption. Deployment in real-world IoT systems and validation of the scheme’s security goals were left for future work. Liu et al. [19] proposed a CapBAC model using decentralized identifiers to support decentralized identity management and secure access control for IoT devices. The model provides tamper-proof access records and is suitable for resource-constrained IoT environments. More recently, Zhu et al. [20] proposed G-CapBAC, a Group-Capability-Based Access Control scheme with ring signature for Smart Campus IoT. Their scheme uses blockchain as a trust anchor and manages capability tokens at the group level to reduce storage requirements and improve access request processing. Ring signatures support group-based authentication, while IoT gateways act as blockchain nodes to avoid placing blockchain overhead on resource-constrained IoT devices. Experimental results showed that G-CapBAC improves storage efficiency and authentication speed compared with traditional CapBAC. However, the performance and security implications in real-world deployments were not fully addressed in either study.

Agostinelli et al. [24] proposed B-CONFIDENT to preserve business confidentiality in coopetitive supply chains. Implemented on GoQuorum, the approach uses smart contracts and access permissions to restrict sensitive transaction data to authorized participants. Its focus is selective disclosure of business data among organizations that both cooperate and compete. The present framework instead focuses on authorizing access to IoT devices: ABAC determines the initial access decision in Hyperledger Fabric, while reusable signed capability tokens support subsequent enforcement at the Smart Gateway. Ledger-authoritative token revocation and event-driven caching support the management and local validation of these tokens. The distinction therefore concerns the protected resources and the authorization workflow, rather than the use of a permissioned blockchain alone.

Overall, existing studies show that blockchain can improve IoT access control by providing decentralization, transparency, immutability, and automated policy enforcement through smart contracts. Hyperledger Fabric is commonly used in permissioned IoT environments because of its modular architecture, identity management, and support for private and scalable access control. Ethereum is also used in several studies because of its smart contract capabilities.

Previous studies have already combined access-control models for IoT, as illustrated by SUPnP [25], which combines ABAC and CapBAC without using blockchain, and have used blockchain-anchored gateway caching to reuse authorization decisions, as in DGAC-IoT [17]. Against this background, the present study implements and evaluates a specific division of responsibilities on Hyperledger Fabric: the initial ABAC evaluation is performed in chaincode and the complete token state is recorded on the Ledger, while the token claims are signed off-chain to produce a reusable capability token that the Smart Gateway enforces using token state obtained either from the Ledger or from its local cache.

Alternative approaches such as Decentralized Identifiers (DIDs) and OAuth 2.0 have also been explored for IoT identity and access control. DID-based models can reduce single-point-of-failure risks by assigning decentralized identities to users and smart devices and combining them with capability-based access control, while OAuth 2.0 can support delegated authorization and trusted third-party access in healthcare systems integrated with Hyperledger Fabric [26,27]. However, these approaches may still require additional mechanisms for fine-grained policy evaluation, token-state auditability, and efficient revocation management. Therefore, this study focuses on a hybrid ABAC and CapBAC framework using Hyperledger Fabric.

In Table 1, several studies are summarized according to their access control models and blockchain platforms. Most studies focus on ABAC because of its fine-grained access control capabilities, while fewer studies investigate CapBAC or hybrid models. The authors of [14] do not specify the blockchain platform used.

Table 2 provides a summary of various studies on access control models in IoT environments using blockchain technology, highlighting their main findings and specific limitations.

Overall, the reviewed studies show that blockchain-based access control can improve decentralization, transparency, and policy enforcement in IoT environments. However, several recurring limitations remain, particularly the overhead caused by repeated blockchain-based authorization decisions, limited evaluation under concurrent IoT workloads, and insufficient testing in realistic deployment settings. The present study addresses the first two limitations within a controlled prototype: it combines ABAC for initial policy evaluation with CapBAC for reusable signed-token enforcement and evaluates gateway-side validation under concurrent workloads.

Table 3 presents an evidence-based comparison of access control models adapted from [28] and extended with the proposed framework. The criteria used in the comparison are defined as follows. *Scalability* refers to the ability of the model to support large numbers of users, devices, resources, or concurrent requests without excessive authorization overhead. *Flexibility* refers to the ability to support dynamic and fine-grained access decisions based on roles, attributes, permissions, conditions, or tokens. *Lightweight enforcement* refers to the per-request authorization overhead, particularly whether access can be checked using role lookup, policy evaluation, or token verification.

According to [28], RBAC has limited scalability in large IoT environments because predefined roles may become difficult to manage as the number of devices and permissions increases. ABAC provides higher scalability and flexibility because access decisions are based on attributes; however, it still requires policy and attribute evaluation during authorization. CapBAC is considered lightweight because it relies on communicable and unforgeable capability tokens, although token management may become more complex in large systems. The proposed ABAC–CapBAC framework extends these models by using ABAC for the initial attribute-based authorization decision and reusable signed CapBAC tokens for subsequent gateway-side validation.

**Table 3:** Comparison of Access Control Models and the Proposed ABAC–CapBAC Framework.

No.	Access Control	Scalability	Flexibility	Lightweight Enforcement
1	RBAC	Limited scalability in large IoT environments because predefined roles for very large numbers of devices may lead to role-assignment complexity and role explosion.	Moderate flexibility because permissions depend mainly on predefined roles.	Moderate enforcement overhead based on role checking, but limited support for dynamic and fine-grained decisions.
2	ABAC	Scalable because access policies are defined using attributes, making it more suitable for complex and granular IoT systems.	High flexibility because decisions can use user, subject, resource, and environmental attributes.	Moderate lightweight enforcement because each request may require policy and attribute evaluation.
3	CapBAC	Scalable through token-based authorization, since token management can be efficient; however, complex systems may require users to manage many tokens.	High flexibility because capability tokens can encode access rights and conditions.	Low enforcement overhead because access can be checked through a communicable and unforgeable token of authority.
4	Proposed ABAC–CapBAC	Reusable signed tokens avoid repeated ABAC evaluation, and cache hits avoid repeated Ledger retrieval; horizontal scalability was not evaluated, as the prototype uses one Smart Gateway instance with a single worker and a process-local cache.	Supports ABAC policy evaluation, including an optional access-time condition, and token-based constraints such as device, action, expiry, and token status.	Cache-assisted Gateway validation reduces repeated Ledger queries and supports efficient signed-token validation.

*Note:* RBAC, ABAC, and CapBAC characteristics are adapted from Table 1 in [28]. The proposed ABAC–CapBAC row is added by the authors based on the design characteristics of the proposed framework.

Table 3 compares the design characteristics of the access-control models and the proposed framework. In the proposed framework, ABAC is used for the initial attribute-based authorization decision, while CapBAC enables subsequent requests to be validated using reusable signed tokens. Separately, the performance comparison in Table 7, Table 8 and Table 9 evaluates the benefit of retrieving authoritative token state from the synchronized cache rather than through a Ledger query for each request, with both modes using the same signed-token validation checks. Based on the reviewed studies, several research gaps can be identified. First, most of the blockchain-based frameworks summarized in Table 1 use a single access-control model, although hybrid designs have also been reported. For example, the framework in [21] combines ABAC and RBAC on Ethereum, whereas SUPnP [25] combines ABAC and CapBAC without using blockchain. The present study examines a specific implementation of ABAC–CapBAC on Hyperledger Fabric. Second, several studies rely mainly on repeated blockchain or smart-contract interactions during access control, which may increase per-request authorization overhead in resource-constrained IoT environments. This study addresses this issue by returning a full signed capability token to the user and enabling gateway-side validation with event-driven cache support. Third, token-state management and revocation handling are not always clearly integrated into the enforcement process. The proposed framework records token state on the ledger and synchronizes issuance and revocation updates through blockchain events. Finally, several studies provide only limited evaluation in permissioned consortium settings. The present study evaluates a controlled, single-host Hyperledger Fabric proof-of-concept with two organizations, a shared endorsement policy, and Ledger-versus-Cache testing; it does not represent a geographically distributed consortium deployment.

The proposed ABAC–CapBAC framework combines attribute-based initial authorization with reusable capability tokens for subsequent access requests. In the current prototype, ABAC supports initial authorization based on the user’s role and department, the device ID, the requested action, and an optional access-time condition. Each capability token is digitally signed using Ed25519. Verification using the trusted Signer’s public key provides assurance of the token’s origin and detects any modification to the signed payload after signing [29]. Hyperledger Fabric provides the permissioned infrastructure for chaincode-based policy evaluation, identity management, and tamper-resistant records. In the proposed framework, the Ledger maintains the authoritative token state, while the Smart Gateway enforces access decisions for subsequent token-based requests without repeating the initial ABAC evaluation.

## 3. Methodology

**Figure 2:**
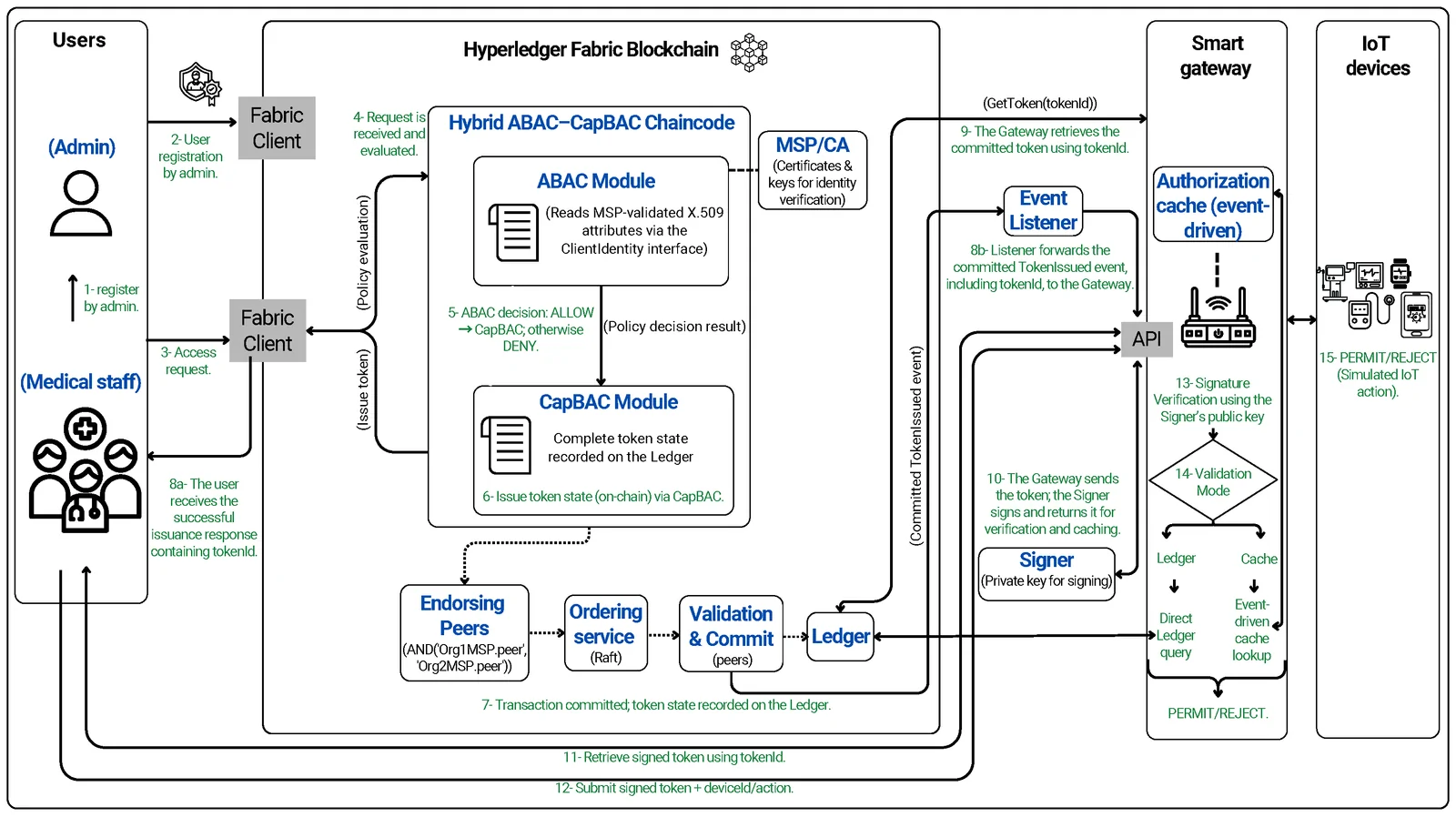
Methodology workflow. Attribute-Based Access Control (ABAC); Application Programming Interface (API); Certificate Authority (CA); Capability-Based Access Control (CapBAC); Internet of Things (IoT); Membership Service Provider (MSP).

This study follows a design, implementation, and evaluation approach to develop and assess a hybrid IoT access-control framework. The framework combines Attribute-Based Access Control (ABAC) and Capability-Based Access Control (CapBAC) using Hyperledger Fabric. ABAC and CapBAC are implemented as two logical modules within a single Hybrid ABAC–CapBAC Chaincode. The framework is designed to combine attribute-based initial policy evaluation with reusable signed capability-token enforcement. The methodology covers authorization, on-chain token-state creation, off-chain token signing, Gateway enforcement, and experimental evaluation, as illustrated in Figure 2.

### 3.1. Design Rationale

The integration of ABAC and CapBAC using Hyperledger Fabric was motivated by the need to balance authorization flexibility, security, and repeated-access efficiency in IoT environments. The ABAC module performs the initial authorization decision using certificate-derived attributes, including role and department, together with the requested device and action. Following an ALLOW decision, the CapBAC module creates an on-chain token state representing the granted capability. After the transaction is successfully committed, selected authorization claims are signed off-chain using Ed25519 [30] to produce a reusable signed capability token.

The Smart Gateway verifies the signature on every request and obtains the current token state either directly from the Ledger or from the synchronized Ledger-derived Cache, while the Ledger remains the authoritative source. Cache-assisted validation reduces repeated Ledger queries without omitting signature verification or token-constraint checks.

Hyperledger Fabric was selected because of its permissioned architecture and certificate-based Membership Service Provider (MSP) identity model [31], as well as its smart-contract support, distributed Ledger, and configurable endorsement policies [32,33]. The ordering service establishes a strict transaction order and packages transactions into blocks for validation and commitment [34]. Together, these mechanisms support authenticated participation, policy-governed transaction validation, and an auditable, tamper-resistant record of access policies, token states, and revocation decisions across the participating organizations.

### 3.2. System Architecture

The proposed framework comprises users and administrators, Fabric Certificate Authority (CA) and MSP, a Fabric Client, the Hyperledger Fabric network, a single Hybrid ABAC–CapBAC Chaincode, an off-chain Event Listener and Signer, a Smart Gateway Application Programming Interface (API), an event-driven authorization Cache, and simulated IoT devices. Within the hybrid chaincode, the ABAC Module performs policy evaluation, while the CapBAC Module manages capability-token state creation, lookup, and revocation.

A user submits an access request through the Fabric Client. The endorsing peers execute the hybrid chaincode according to the AND(‘Org1MSP.peer’,‘Org2MSP.peer’) endorsement policy. The ABAC Module reads the authenticated subject, MSP identifier, role, and department from the MSP-validated X.509 identity, and evaluates the role and department together with the requested device and action. If the policy decision is ALLOW, the CapBAC Module creates the token state and prepares a TokenIssued event within the same transaction. The token state becomes authoritative only after successful endorsement, ordering, validation, and commit to the Ledger.

After commit, the client receives the successful issuance response containing tokenId, while the committed TokenIssued event is delivered to the Event Listener and forwarded to the Smart Gateway. Using tokenId, the Gateway retrieves the committed token state from the Ledger and sends it to the off-chain Signer. The Signer signs the selected authorization claims using its Ed25519 private key and returns the signed token to the Gateway. The Gateway verifies the signature using the corresponding pre-provisioned Signer public key, compares the signed claims with the committed Ledger state, and stores a synchronized Ledger-derived copy of the token state and the verified signed token in its local Cache. The user subsequently retrieves the signed token from the Gateway using tokenId.

The Smart Gateway serves as the enforcement point between users and the simulated IoT environment. For each access request, the user submits the signed token together with the currently requested device and action. The Gateway first verifies the Ed25519 signature and then obtains the authoritative token state through the selected validation mode. Ledger mode performs a direct Ledger query, whereas Cache mode uses the event-driven local Cache without a per-request Ledger query on a cache hit. The Gateway validates the authoritative status, expiry, requested device, and requested action and compares the signed claims with the authoritative state before returning PERMIT or REJECT. A permitted IoT action is simulated in the current prototype.

### 3.3. Methodology Workflow

The workflow begins with user registration and certificate-based identity provisioning through Fabric CA and MSP. Administrative identities also establish the required ABAC policies on the Ledger. When a user submits an access request, the ABAC Module evaluates the certificate-derived role and department together with the requested device and action. If the policy is not satisfied, the request is denied, and no token state or TokenIssued event is produced.

If the policy is satisfied, the CapBAC Module creates an on-chain token state containing tokenId, tokenRef, subject, MSP identifier, role, department, device, action, expiry, and status. The transaction is then endorsed, ordered, validated, and committed. Following successful commit, the user receives an issuance response containing tokenId, and the committed TokenIssued event triggers the off-chain signing workflow. The Gateway retrieves the committed state, the Signer signs the selected authorization claims, and the Gateway verifies and caches the resulting signed token. The user then retrieves the signed token from the Gateway using tokenId.

For a subsequent access attempt, the user submits the signed token, requested device, and requested action to the Gateway. Signature verification occurs before authoritative token-state validation. The Gateway then uses either Ledger mode or Cache mode to retrieve the authoritative state. It checks that the token is active and unexpired, verifies the device and action, and confirms that the signed claims match the authoritative token state. If all checks succeed, the Gateway returns PERMIT and simulates the requested IoT action; otherwise, it returns REJECT. Committed TokenIssued and TokenRevoked events keep the Gateway Cache synchronized with the Ledger state.

The complete methodology workflow is illustrated in Figure 2.

### 3.4. Sequence Diagrams

**Figure 3:**
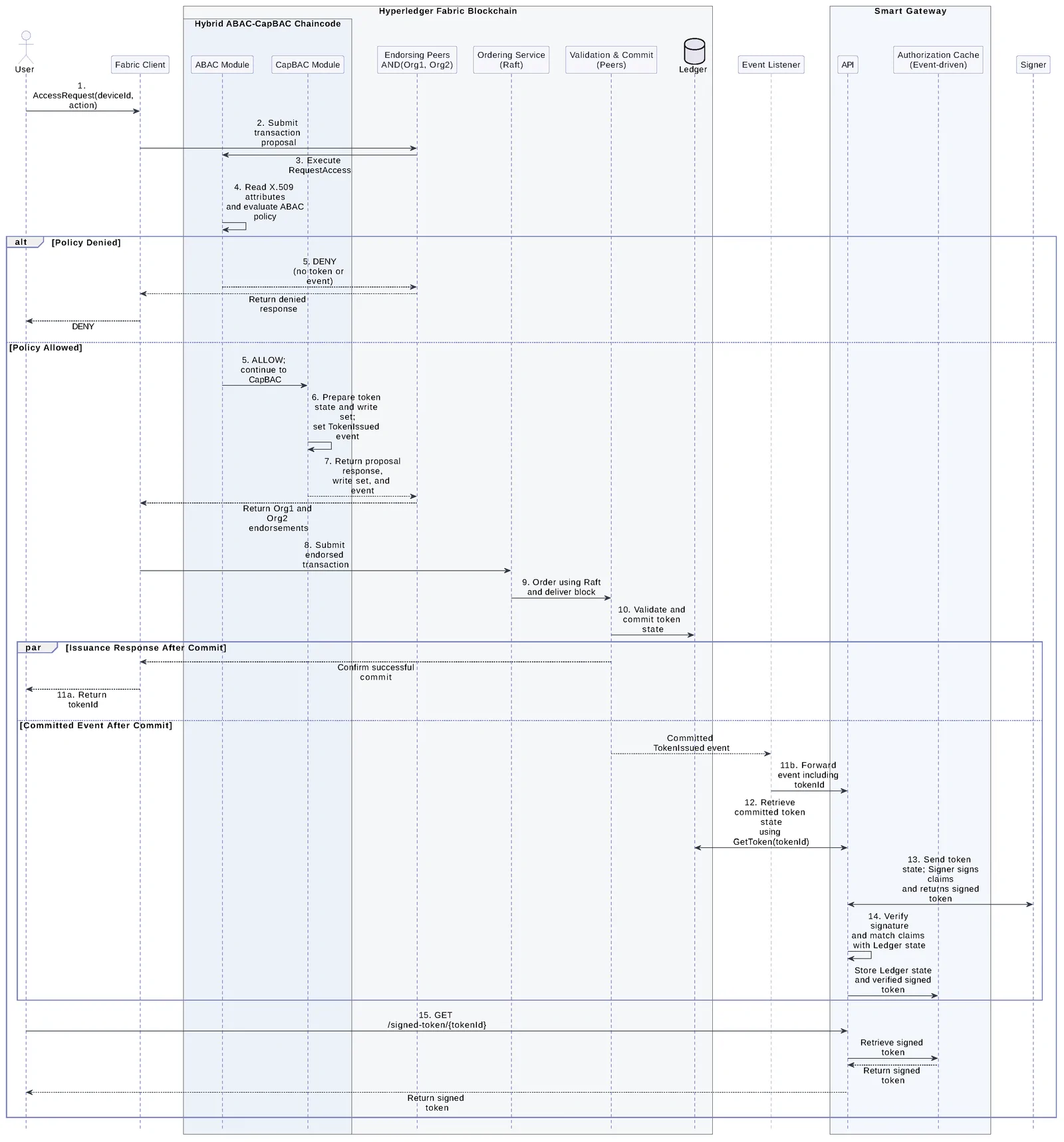
Sequence diagram of the signed capability-token issuance workflow.

**Figure 4:**
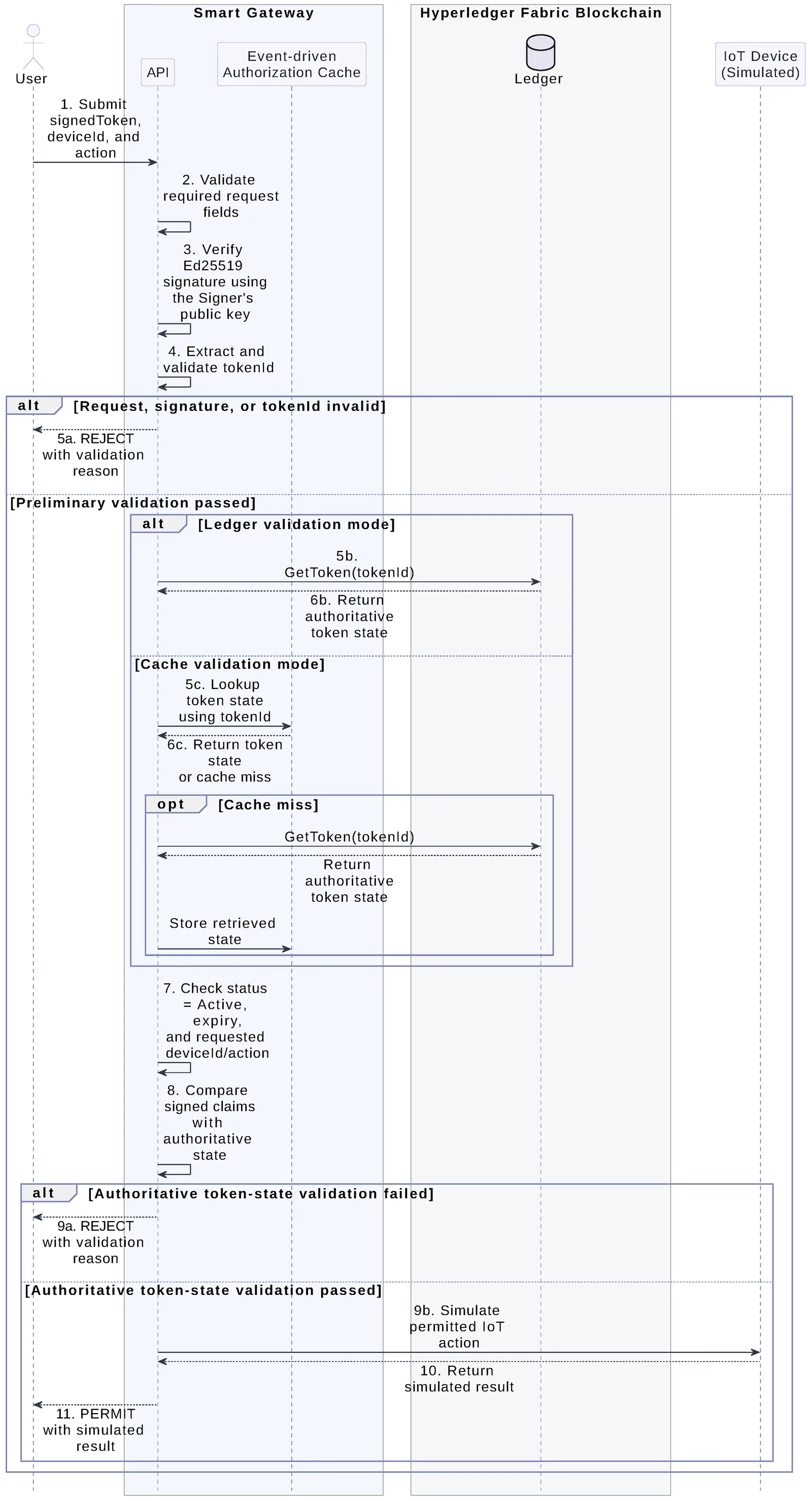
Sequence diagram of the access validation workflow.

Figure 3 and Figure 4 show the component interactions during token issuance and subsequent access validation, respectively.

Figure 3 highlights the separation between on-chain token-state creation and off-chain token signing. The ABAC and CapBAC functions are implemented as logical modules within a single hybrid chaincode. Following an ALLOW decision, the token state becomes authoritative only after endorsement, ordering, validation, and successful commit. The committed TokenIssued event then triggers off-chain signing, verification, and caching at the Smart Gateway, after which the user retrieves the signed token using the returned tokenId.

Figure 4 shows that the Smart Gateway first verifies the Ed25519 signature using the Signer’s public key and validates the tokenId. The Gateway then retrieves the authoritative token state using either a direct Ledger query or the event-driven authorization cache, with a Ledger fallback on a cache miss. The Gateway subsequently checks the current token status, expiry, requested device and action, and matches the signed claims against the authoritative state. If all checks succeed, the requested IoT action is permitted and simulated in the prototype; otherwise, the request is rejected.

### 3.5. Revocation Propagation and Cache Consistency

In this paper, token state denotes the complete on-chain token record, whereas token status denotes its Active/Revoked field. The Ledger remains authoritative for token state. The Gateway cache holds a local copy that is updated through committed chaincode events; these updates are asynchronous and therefore do not provide immediate consistency with the Ledger.

When an administrator invokes RevokeToken, the chaincode updates the token status and emits a TokenRevoked event within the same transaction. Once the transaction has been validated and committed, the Event Listener forwards the committed event to the Smart Gateway. The Gateway changes the status of an existing cached entry to Revoked and removes the corresponding cached signed token. Ledger-mode validation can retrieve the revoked status after commit on the queried peer. In Cache mode, an existing entry may remain Active until the event has been applied, allowing an otherwise valid signed token to be accepted during this interval. A cache miss instead triggers retrieval of the token state from the Ledger. Section 4.3.10 quantifies this interval under normal, delayed, and interrupted event delivery.

For missed-event recovery, the Listener maintains a persistent checkpoint and advances it only after the Gateway returns a successful Hypertext Transfer Protocol (HTTP) response. If event processing fails, the Gateway returns HTTP 503, and the checkpoint is not advanced. The Listener reconnects and resumes event delivery from the saved checkpoint, which is also used when the Listener restarts. This checkpoint-based event replay resynchronizes the cache with committed events rather than assuming that every event is processed successfully on its first delivery.

Checkpoint persistence and event replay are implemented in the prototype. Their evaluation was limited to five trials in which the Listener was stopped and restarted during the revocation experiment. These trials provide evidence of recovery after the tested interruption, but do not validate the recovery mechanism under other failure conditions. The HTTP 503 non-acknowledgment and subsequent event-redelivery path was not evaluated separately, and the handling of old or duplicate events was not evaluated.

For a TokenIssued event, the Gateway retrieves the current token state from the Ledger rather than treating the event payload as the complete record. If the retrieved status is no longer Active, the Gateway caches that state and removes any cached signed token without requesting a new signature. The signing and caching workflow is described in Section 3.6.2, and the validation checks are described in Section 3.6.3.

Changes to a user’s role or department do not automatically update previously issued capability tokens. Each token records the role and department under which it was issued, and the Gateway validates the signed claims against the stored token state rather than against the user’s current attributes. An active token issued under the previous attributes therefore remains valid until it expires or is explicitly revoked. To enforce an attribute change, the administrator must explicitly revoke each affected token through RevokeToken. The user must then submit a new access request, which undergoes a new ABAC evaluation using the attributes presented by the client identity at request time (Section 3.6.1) before a new token can be issued. The same limitation applies to policy changes: the chaincode does not allow an existing policy to be modified or deleted, and the Gateway does not re-evaluate policies during token validation. Consequently, a policy added after token issuance does not affect tokens that are already active.

### 3.6. Pseudocode

The proposed framework uses three main algorithms: Hybrid ABAC–CapBAC authorization and token-state creation, event-driven off-chain signed-token generation and caching, and gateway-side signed-token validation and access enforcement.

#### 3.6.1. Hybrid ABAC–CapBAC Authorization and Token-State Creation

In the proposed framework, RequestAccess is executed by a single Hybrid ABAC–CapBAC Chaincode containing two logical modules. The ABAC module retrieves the authenticated subject, MSP identifier, role, and department from the transaction’s MSP-validated X.509 client identity through Fabric’s ClientIdentity interface [31,35]. The ABAC module evaluates the certificate-derived role and department together with the requested deviceId and action. A policy created through AddTimePolicy also specifies an absolute Coordinated Universal Time (UTC) access window, [accessStart, accessEnd), which is evaluated against the Fabric transaction timestamp. Policies created through AddPolicy have no time restriction. The chaincode examines policies in Ledger key order and applies the effect of the first policy for which all specified conditions are satisfied; if no policy matches, the decision is DENY. If the result is not ALLOW, the request is denied without creating a token state or emitting a TokenIssued event.

A new access request is evaluated using the role and department contained in the client certificate presented with that request. Updated attributes therefore affect the decision only when the presented certificate contains those values, as discussed in Section 3.5.

Following an ALLOW decision, the CapBAC module creates the unsigned token state and invokes PutState to add the proposed state update to the transaction write set. It also invokes SetEvent to attach a reduced TokenIssued event to the transaction proposal response [36]. No Ledger update occurs during endorsement simulation. The proposed state update becomes authoritative only after the transaction is endorsed, ordered, validated as VALID, and committed [37,38]. The associated TokenIssued event is processed by the off-chain components only after successful commit confirmation [39]. The client then receives the successful response containing the token state, including tokenId, while the Event Listener receives the committed event. Token signing is performed subsequently by the off-chain Signer and is therefore outside Algorithm 1.
**Algorithm 1** RequestAccess: Hybrid ABAC–CapBAC Authorization and Token-State Creation**Require:** deviceId, action, authenticated Fabric transaction context ctx**Ensure:** Access decision and token state if authorized  1:userIdentity← GetClientIdentity(ctx)  2:subject← GetID(userIdentity)  3:mspID← GetMSPID(userIdentity)  4:role← GetAttributeValue(userIdentity, role)  5:department← GetAttributeValue(userIdentity, department)  6:**if** role or department is unavailable **then**  7:      **return** ERROR  8:**end if**  9:txTime← GetTransactionTimestamp(ctx)10:(effect,accessEnd)← EvaluatePolicyAt(ctx, role, department, deviceId, action, txTime)*A time-bounded policy matches only when accessStart≤txTime<accessEnd;**policies created through* *AddPolicy* *have no time condition.*11:**if** effect≠ALLOW **then**12:      **return** DENY, Access denied due to policy mismatch13:**end if**14:txId← GetTransactionID(ctx)15:tokenSeed←subject∥NUL∥deviceId∥NUL∥action∥NUL∥txId*where NUL denotes the single zero-byte separator used in the implementation.*16:tokenId←TOKEN_∥HEX(SHA256(tokenSeed))17:tokenRef←REF_∥tokenId18:expiry←txTime+1hour19:**if** accessEnd is present and accessEnd<expiry **then**20:      expiry←accessEnd21:**end if**22:status←Active23:tokenState←{tokenId,tokenRef,subject,mspID,                role,department,deviceId,action,expiry,status}24:eventPayload←{TokenIssued,tokenId,subject,                deviceId,action,status,txTime}25:PutState(tokenId, tokenState)26:SetEvent(TokenIssued, eventPayload)27:response←{ALLOW,Access granted and token issued,tokenState}*The state write and event become authoritative and observable**only after Fabric commits the transaction asVALID*.28:**return** response

#### 3.6.2. Event-Driven Signed Token Generation

Algorithm 2 describes the signing and caching path for a token whose retrieved Ledger status is Active, after successful commit of the transaction that emits the TokenIssued event. The handling of non-active token state is described in Section 3.5. The Event Listener forwards the committed event to the Gateway API. Using the tokenId contained in the event, the Gateway retrieves the complete authoritative token state from the Ledger through GetToken(tokenId); the event payload itself is not treated as the complete token state.

The Gateway sends the committed token state to the off-chain Signer, which signs the selected authorization claims using its Ed25519 private key and returns the signed token to the Gateway. The auxiliary tokenRef is not signed because it is not used in access-validation decisions. The Gateway verifies the signature using the pre-provisioned Signer public key, confirms that the signed claims match the Ledger state, and then caches the authoritative state and signed token. The user subsequently retrieves the signed token from the Gateway using tokenId.

The signed status represents an issuance-time snapshot. During access validation, the current authoritative token state is obtained from the Ledger or its synchronized event-driven Cache.
**Algorithm 2** Event-Driven Off-Chain Signed Token Generation and Caching**Require:** Committed chaincode event, Signer-held privateKey, Gateway-provisioned publicKey**Ensure:** Verified signed token cached at the Gateway  1:(eventName,eventPayload)← EventListenerReceiveCommittedEvent()  2:**if** eventName≠TokenIssued **then**  3:      **return** NO_SIGNING_ACTION  4:**end if**  5:EventListenerForwardToGateway(eventName, eventPayload)  6:tokenId←eventPayload.tokenId  7:**if** IsValidTokenID(tokenId) =false **then**  8:      **return** ERROR  9:**end if**10:tokenState← Ledger.GetToken(tokenId)11:**if** tokenState does not exist **then**12:      **return** ERROR13:**end if**14:GatewaySendToSigner(tokenState)15:signedFields←{tokenId,subject,mspId,role,department,                    deviceId,action,expiry,status}16:**if** any field in signedFields is missing from tokenState **then**17:      **return** ERROR18:**end if**19:payload← SelectFields(tokenState, signedFields)20:serializedPayload← CanonicalJSON(payload)21:signatureBytes← Ed25519Sign(privateKey, serializedPayload)22:signature← Base64Encode(signatureBytes)23:signedToken←{payload,signature,alg=Ed25519}24:SignerReturnToGateway(signedToken)25:**if** Ed25519Verify(publicKey, signature, serializedPayload) =false **then**26:      **return** ERROR27:**end if**28:**if** SignedFieldsMatch(payload, tokenState) =false **then**29:      **return** ERROR30:**end if**31:GatewayCacheTokenState(tokenId, tokenState)32:GatewayCacheSignedToken(tokenId, signedToken)*The signed token is available for subsequent retrieval using tokenId.*

#### 3.6.3. Signed-Token Validation and Access Enforcement

Algorithm 3 validates the signed capability token and enforces the access decision at the Smart Gateway. The selected endpoint determines whether the authoritative token state is obtained through Ledger mode or Cache mode, while both endpoints use the same validation flow.

The Gateway first verifies the Ed25519 signature using the pre-provisioned Signer public key and validates tokenId. It then retrieves the token state directly from the Ledger or from the synchronized event-driven Cache, with Ledger fallback on a Cache miss. The Ledger remains the authoritative source of token state.

The Gateway verifies the token status, expiry, requested device, and action, and confirms that the signed authorization claims match the retrieved state. The auxiliary tokenRef is excluded because it is not a signed authorization claim. If all checks succeed, access is permitted and the prototype returns a simulated IoT execution result.

For compactness, Algorithm 3 uses four abstract operations corresponding to the implemented Gateway logic. VerifySignedToken represents signed-token format validation and Ed25519 signature verification over the canonical JavaScript Object Notation (JSON) payload. GetAuthoritativeToken represents a direct Ledger query in Ledger mode, or an event-driven Cache lookup with Ledger fallback in Cache mode. SignedFieldsMatch compares the nine signed authorization fields with the retrieved authoritative token state. Finally, SimulateIoTAction represents the simulated IoT execution result returned by the prototype.
**Algorithm 3** Signed-Token Validation and Access Enforcement at the Smart Gateway**Require:** signedToken, requestedDeviceId, requestedAction, mode∈{ledger,cache}, publicKey**Ensure:** PERMIT or REJECT  1:**if** VerifySignedToken(signedToken, publicKey) =false **then**  2:      **return** REJECT, Invalid token signature  3:**end if**  4:payload←signedToken.payload  5:tokenId←payload.tokenId  6:**if** IsValidTokenID(tokenId) =false **then**  7:      **return** REJECT, Missing or invalid tokenId in signed payload  8:**end if**  9:tokenState← GetAuthoritativeToken(tokenId, mode)10:**if** tokenState=NULL **then**11:      **return** REJECT, Token lookup failed12:**end if**13:**if** tokenState.status≠Active **then**14:      **return** REJECT, Token is not active15:**end if**16:**if** CurrentUTCTime() >tokenState.expiry **then**17:      **return** REJECT, Token expired18:**end if**19:**if** tokenState.deviceId≠requestedDeviceId **or** tokenState.action≠requestedAction **then**20:      **return** REJECT, Device or action mismatch21:**end if**22:**if** SignedFieldsMatch(payload, tokenState) =false **then**23:      **return** REJECT, Signed payload mismatch24:**end if**25:result← SimulateIoTAction(requestedDeviceId, requestedAction)26:**return** PERMIT, result

### 3.7. On-Chain and Off-Chain Design

A central design decision in the proposed framework is the separation of on-chain and off-chain operations. Security-critical and auditable data are managed by the single Hybrid ABAC–CapBAC Chaincode and stored on the Hyperledger Fabric ledger. These data include ABAC policies and capability-token states, including the token status used to record revocation. User attributes, such as role and department, are obtained from the MSP-validated X.509 certificate and evaluated inside the chaincode; they are not maintained as separate identity records on the ledger, although selected attribute values are included in the issued token state.

Performance-sensitive and cryptographic operations are performed off-chain. These operations include Ed25519 token signing, signed-token handling, committed-event listening, gateway-side signature verification, authorization-cache management, and access enforcement. The full signed token is not stored on the Ledger. In the current prototype, the resulting IoT operation is simulated rather than executed on a physical IoT device.

Table 4 summarizes the placement and rationale of the main framework components.

### 3.8. Key Management and Signer Trust Model

During prototype setup, a separate Ed25519 key pair is generated off-chain for capability-token signing and verification. Consistent with the Ed25519 signing model, the private key is retained by the Signer to generate token signatures, while the corresponding public key is pre-provisioned at the Smart Gateway to verify those signatures [29,30]. This application-level Ed25519 key pair is separate from the X.509 enrollment identity used by Fabric’s CA/MSP-based identity infrastructure [31].

Following successful commit confirmation for a transaction that emits a TokenIssued event, the Gateway retrieves the authoritative token state from the Ledger and sends it to the Signer [38,39]. The Signer signs the selected token claims and returns the resulting signed token to the Gateway, which verifies and caches it together with the corresponding Ledger-derived token state.

The prototype treats the Signer as a trusted off-chain component. During token validation, a valid signature alone is insufficient; the Gateway also compares the signed claims with the Ledger-authoritative token state obtained either through a direct Ledger query or from the event-synchronized cache.

### 3.9. Threat Model

The proposed framework considers an IoT access-control environment in which users, gateways, and IoT devices communicate over a network that may be exposed to external attackers. The attacker may be an external network adversary who attempts to intercept, modify, replay, or inject access requests; a malicious insider who owns valid system credentials but attempts to access unauthorized devices or perform unauthorized actions; or an attacker who obtains an old or revoked token and attempts to reuse it. The model also considers the risk of stale Gateway Cache state during delayed event synchronization, where an attacker may attempt to exploit an outdated Active token status.

The framework is designed to reject unauthorized access, token forgery, token tampering, wrong-device or wrong-action token reuse, expired-token usage, and revoked-token reuse when the Gateway has synchronized token state or confirms the state directly from the Ledger. These protections are provided through ABAC-based policy evaluation, capability-token issuance only after authorization, Ed25519 signature verification, device–action binding, expiry checking, authoritative token-status validation, and Ledger-backed auditability. Token expiry limits the validity period during which a captured token may be reused, while token-state validation rejects it after expiration or revocation.

The off-chain Signer is part of the trusted computing base. In the current prototype, the Signer loads its Ed25519 private key from a local, unencrypted Privacy-Enhanced Mail (PEM) file, while the Smart Gateway holds the corresponding public key for signature verification. Compromise of the private key could allow an attacker to produce cryptographically valid signatures. The Gateway additionally compares the signed claims with the authoritative token state and checks the status, expiry, device, and action. These checks limit the acceptance of claims that are inconsistent with the recorded token state, but they do not eliminate the risk of Signer-key compromise; in Cache mode, they also depend on the freshness of the cached state. Production-grade key storage, key rotation, and compromise recovery are not implemented or evaluated.

The following attacks are outside the scope of the current prototype: physical compromise of IoT devices, hardware tampering, denial-of-service attacks, full compromise of the Fabric CA/MSP, compromise of the off-chain Signer or its private key, and full compromise of the Smart Gateway execution environment. In particular, the Gateway is assumed to execute the validation logic correctly. If the Gateway is fully compromised, an attacker may bypass local enforcement regardless of the access-control model. These issues require additional deployment-level protections such as secure hardware, intrusion detection, rate limiting, Key Management Service-based key protection, threshold signing, and Gateway hardening.

### 3.10. Implementation and Evaluation

The framework was implemented using Hyperledger Fabric chaincode for ABAC evaluation, token-state creation, token retrieval, and token revocation. Following successful transaction commit, the Event Listener triggered off-chain signed-token generation and synchronized the Gateway Cache using committed token events. The Smart Gateway validated signed tokens using either Ledger mode or Cache mode and returned a simulated IoT execution result for permitted requests.

## 4. Results

The proposed framework was evaluated through functional and security validation and a performance comparison between Ledger and Cache validation modes, with additional experiments on multiple users and tokens, cache-hit ratios, emulated network delay, token issuance, TokenIssued synchronization, and revocation propagation. In the main repeated-validation benchmark, the Smart Gateway validated signed capability tokens in both modes. Ledger mode retrieved the authoritative token state from the Ledger through a persistent Fabric Gateway Software Development Kit (SDK) connection, whereas Cache mode used synchronized Ledger-derived token state from the local Cache, which was populated before each timed batch. Performance was evaluated at concurrent request counts of 50, 100, 200, 500, and 1000 using batch completion time (BCT), mean request latency, and throughput.

Batch completion time is the elapsed wall-clock time, in seconds, measured from immediately before launching the batch until all request processes have completed. For each run, mean request latency is the arithmetic mean of the individually measured HTTP request durations, obtained using curl’s time_total [40] and converted to milliseconds. Throughput is calculated by dividing the number of successfully processed requests by the batch completion time in seconds. In the main repeated-validation benchmark, each workload was repeated five times in each mode, and the reported means and sample standard deviations were calculated across these five repetitions.

The results show that Cache mode consistently outperformed Ledger mode across all tested workloads. In Ledger mode, mean BCT increased from 0.25 s at 50 concurrent requests to 4.52 s at 1000 requests. In Cache mode, mean BCT increased from 0.13 s to 2.80 s over the same range. At 1000 concurrent requests, Cache mode reduced mean request latency by 46.61% and achieved 1.62 times the throughput of Ledger mode. At this workload, each of the five timed Ledger-mode batches recorded 1000 Ledger queries and no cache hits, whereas each of the five timed Cache-mode batches recorded no Ledger queries and 1000 cache hits.

### 4.1. Experimental Environment

To support reproducibility, Table 5 summarizes the experimental environment used to implement and evaluate the proposed framework.

Accordingly, the concurrency experiments characterize one Smart Gateway instance running a single worker with a process-local cache; they do not evaluate horizontal scale-out across multiple Gateway instances.

The repeated-validation benchmark was extended to ten Fabric identities, with ten distinct signed tokens issued to each identity. Requests within each batch were assigned across the resulting 100 tokens in a round-robin manner, with equal representation of the ten identities. The experiment used the same workload sizes and five repetitions per validation mode as the single-token benchmark. Before each Cache-mode measurement, the token states required by that batch were loaded into the cache outside the timed interval.

To isolate the effect of cache availability, the cache-hit-ratio experiment used planned cache-hit ratios of 0%, 25%, 50%, 75%, and 100%. Each batch contained 100 concurrent requests, with one request for each of the 100 signed tokens issued to ten Fabric identities. Before every batch, the cache was cleared and the number of token states required by the planned ratio was loaded outside the timed interval. Cache misses were served through the persistent Fabric Gateway connection and populated the cache during the measurement. Each ratio was evaluated over 20 repetitions, and the order of the ratios was randomized within each repetition.

A separate experiment examined the effect of cache capacity while keeping the workload fixed. An experimental Gateway extension provided a least-recently-used (LRU) cache with capacities of 25, 50, 75, and 100 token-state records, together with an unbounded configuration using the same implementation. Capacity was defined by the number of records, rather than memory usage in bytes. This extension was used only for the capacity experiment; the previously reported experiments were not rerun with it.

**Table 5:** Experimental environment.

Item	Configuration
Software platform	64-bit Windows host with Ubuntu 22.04.5 LTS on Windows Subsystem for Linux 2 (WSL2) and deployment using Docker Desktop 4.60.1 (218372).
Hyperledger Fabric version	Hyperledger Fabric v2.5.15.
Fabric CA version	Hyperledger Fabric CA v1.5.17.
Docker Desktop version	4.60.1 (218372).
Network topology	Two organizations, Org1 and Org2, with one peer per organization and one ordering service.
Peers	Two peers: peer0.org1.example.com and peer0.org2.example.com.
Ordering service	One orderer, orderer.example.com, configured as a single-node Raft ordering service.
Channel	One application channel, mychannel.
Endorsement policy	AND(’Org1MSP.peer’,’Org2MSP.peer’).
Deployment model	Local Docker-based deployment using Hyperledger Fabric test network on Ubuntu under WSL2.
Smart contract	Hybrid ABAC–CapBAC Chaincode.
Gateway validation modes	Ledger mode and Cache mode.
Ledger-access implementation	Persistent Fabric Gateway connection using @hyperledger/fabric-gateway v1.7.1, with the same gRPC client and Gateway instance reused for GetToken queries.
Ledger-access runtime	Node.js v18.20.8 and @grpc/grpc-js v1.14.4.
Main and multi-user benchmark workload generator	Custom Bash script launching *N* concurrent curl processes as background jobs and synchronizing on wait; curl 7.81.0.
Main and multi-user benchmark workload sizes	50, 100, 200, 500, and 1000 concurrent requests.
Experimental repetitions and samples	Five runs for each workload size and validation mode in the Ledger–Cache and multi-user benchmarks; 20 repetitions per condition in the cache-hit-ratio, cache-capacity, and network-delay experiments; five measured trials per event-delivery condition in the revocation experiment; and 100 issued tokens in each of the token-issuance and TokenIssued-synchronization experiments.
IoT endpoint	Simulated IoT device endpoint, such as monitor1.
Gateway server profile	Flask 3.1.3 served by Gunicorn 26.0.0: one gthread worker with 64 threads, backlog 2048 and a 300 s timeout. A single worker was used because the cache and the measurement counters are process-local.
Validation-performance metrics	Batch Completion Time, Mean Request Latency, and Throughput.
Hardware specifications	Intel Core i7-8750H CPU, 16 GB RAM.

*Note:* central processing unit (CPU); gRPC Remote Procedure Calls (gRPC); Long-Term Support (LTS); random access memory (RAM).

The workload consisted of 100 signed tokens issued to ten Fabric identities, with ten tokens per identity. Each run comprised 1000 requests issued with a client concurrency of 64. Each token appeared ten times in a shuffled request sequence generated using a fixed seed. Within each repetition, the same submission sequence was used for all capacities, while the order of the capacity conditions was randomized. Each capacity was evaluated over 20 repetitions. Before each run, the cache was cleared and all 100 token states were loaded sequentially outside the timed interval, leaving the cache populated up to its configured capacity.

On a cache miss, the Gateway retrieved the token state through the persistent Fabric Gateway SDK connection and inserted it into the cache, evicting the least-recently-used record when necessary. Token issuance, signing, preflight validation, and cache loading were excluded from the timed interval. Request latency was measured from the start of execution in a client worker until the response had been read, excluding time spent waiting in the client’s executor queue. Batch completion time included request dispatch and completion of all requests. The recorded metrics were batch completion time, mean request latency, throughput, cache-hit ratio, cache misses, evictions, and Ledger-query counts.

Token issuance was measured separately from repeated signed-token validation. The experiment issued 100 tokens sequentially to ten Fabric identities, with ten tokens issued to each identity. Fabric issuance was measured from launching the peer command-line interface (CLI) invocation of RequestAccess until the CLI reported VALID commit confirmation at both peers. The off-chain Signer time was measured through a separate HTTP request to the Ed25519 Signer. The end-to-end issuance measurement included these two stages and the local orchestration between them.

Token-issuance synchronization was measured separately for 100 newly issued tokens belonging to ten Fabric identities. For each token, the experiment recorded the CLI-observed VALID commit-confirmation time for peer0.org1, the peer used by the Event Listener, the time at which the Event Listener received the committed TokenIssued event (eventReceivedAtUtc), and the time at which the Gateway completed processing the event and made the token available in its local cache (cacheReadyAtUtc). Cache readiness indicates that the Gateway had retrieved the committed token state from the Ledger, obtained and verified the signed token, and cached both records. No artificial event-forwarding delay, cache pre-warming, or fixed waiting interval was applied during this measurement.

### 4.2. Functional and Security Validation

Section 4.2 and Table 6 address RQ1 by presenting the functional and security validation results.

Functional and security validation demonstrated that the framework correctly handled the tested access-control operations. Authorized requests were granted and resulted in token issuance, whereas unauthorized requests were denied according to the ABAC policy rules. The framework also correctly supported token retrieval by tokenId, alternative lookup by tokenRef, event-driven token signing and caching, and administrator-controlled token revocation. Valid signed-token requests were successfully permitted through both Ledger and Cache validation modes and produced the expected simulated IoT execution result.

From a security perspective, the Smart Gateway rejected tampered signatures, malformed token identifiers, expired and revoked tokens, signed claims that did not match the authoritative Ledger state, and requests containing an incorrect device or action. These results indicate that, under the tested scenarios, the framework enforces token integrity, validity, revocation status, device–action binding, and consistency between the signed claims and token state through Ed25519 signature verification, constraint validation, and comparison with either the authoritative Ledger state or its synchronized Ledger-derived Cache copy. Table 6 summarizes the functional and security validation results.

As shown in Table 6, all validation cases passed successfully. Under the tested scenarios, the results demonstrate that the proposed framework correctly supports ABAC authorization, token issuance and retrieval, event-driven signing and cache synchronization, administrator-controlled revocation, and the rejection of invalid signed-token access attempts.

**Table 6:** Summary of Functional and Security Validation Results.

Test	Scenario	Expected Result	Actual Result	Status
T1	Doctor requests control on monitor1	Allow	ALLOW; an active token was issued for doctor control on monitor1.	Pass
T2	Nurse requests read on monitor1	Allow	ALLOW; an active token was issued for nurse read on monitor1.	Pass
T3	Nurse requests control on monitor1	Deny	DENY; access was denied due to policy mismatch, and no token was issued.	Pass
T4	GetToken returns tokenRef	Matching reference returned	The token record was returned with the matching tokenRef.	Pass
T5	GetTokenByRef returns the matching token	Matching token returned	The same token was retrieved successfully using tokenRef.	Pass
T6	TokenIssued event handled	Token state cached	Token state was cached after token issuance.	Pass
T7	TokenRevoked event handled	Cached status updated	Cached token status changed to Revoked.	Pass
T8	Admin revokes the doctor token using tokenId	Status becomes Revoked	The transaction was committed as valid, and the authoritative token status became Revoked.	Pass
T9	Tampered signed token	Reject	REJECT; Invalid token signature.	Pass
T10	Invalid tokenId format in a validly signed payload	Reject	REJECT; Missing or invalid tokenId in signed payload.	Pass
T11	Device mismatch	Reject	REJECT; Device or action mismatch.	Pass
T12	Action mismatch	Reject	REJECT; Device or action mismatch.	Pass
T13	Signed payload and authoritative Ledger-state mismatch	Reject	REJECT; Signed payload mismatch in field: department.	Pass
T14	Reuse of a revoked signed token through Cache	Reject	REJECT; Token is not active.	Pass
T15	Expired signed token	Reject	REJECT; Token expired.	Pass
T16	Doctor requests read within the policy’s access-time window	Allow	ALLOW; an active token was issued, with expiry limited to the window end (accessEnd).	Pass
T17	Doctor requests read after the policy’s access-time window	Deny	DENY; access was denied due to policy mismatch, and no token was issued.	Pass

### 4.3. Performance Evaluation

Section 4.3 and Table 7, Table 8 and Table 9 address RQ2 by comparing the performance of repeated signed-token validation in Cache and Ledger modes under increasing concurrent workloads.

The main repeated-validation benchmark compared two validation modes: Ledger and Cache. In both modes, the Smart Gateway verified the Ed25519 signature on every request and compared the signed authorization claims with the authoritative token state. The difference between the two modes was the source used to retrieve that state. Ledger validation retrieved the authoritative token state through a persistent Fabric Gateway SDK connection for every request. Before the timed experiment began, the gRPC Remote Procedure Calls (gRPC) client and Fabric Gateway instance were initialized once, and gRPC connection readiness was confirmed. The same client and Gateway instance were reused for all GetToken queries across all Ledger-mode workloads and repetitions. Cache validation used the locally synchronized authorization cache and performed no per-request Ledger query on a cache hit.

The main repeated-validation benchmark used concurrent request counts of 50, 100, 200, 500, and 1000. Each request count was repeated five times for each validation mode. Every measured request completed successfully. The reported values represent the mean ± sample standard deviation (SD) across the five repetitions.

Before the benchmark began, the initial ABAC evaluation, token issuance, TokenIssued event processing, off-chain signing, and retrieval of the resulting signed token were completed. Before each timed repetition, the script reset the Gateway statistics and authoritative-state cache while preserving the signed token. In Cache mode, the script then repopulated the authoritative-state cache through one untimed Ledger query using the /warm-cache endpoint. One untimed preflight validation request was subsequently executed in both modes, after which the measurement counters were reset without altering the prepared cache state. Therefore, the timed interval covered only the concurrent repeated signed-token validation requests, and the reported Cache results represent steady-state cache validation.

The performance metrics used in the experiment are defined as follows.

*Batch Completion Time (BCT)* refers to the total batch wall-clock time, measured in seconds, required to complete all requests at a given concurrent request count. The timer was started immediately before the client began launching the concurrent curl processes and stopped after all launched processes had completed. Consequently, BCT includes client-side process-launch and scheduling overhead within the measured batch interval. This interval is conceptually similar to the total-test interval reported by ApacheBench and the first-to-last-sample interval used by JMeter for throughput calculation, although the exact timing boundaries differ [41,42]. BCT was calculated as shown in Equation (1).

(1) $$\mathrm{BCT}=t_{\mathrm{end}}-t_{\mathrm{start}}$$

*Mean Request Latency* is the arithmetic mean of the individually measured HTTP request durations in each run, obtained using curl’s time_total [40] and converted to milliseconds, as shown in Equation (2).

(2) $$\begin{array}{cc} L_{i} & =\left(t_{\mathrm{res},i}-t_{\mathrm{start},i}\right)\times 1000,\\ \bar{L} & =\frac{1}{N_{s}}\sum_{i=1}^{N_{s}}L_{i} \end{array}$$

Here, $N_{s}$ is the number of successfully processed requests in the run. The times $t_{\mathrm{start},i}$ and $t_{\mathrm{res},i}$ denote the start of the individual transfer and receipt of its complete response, respectively, and are expressed in seconds. Both $L_{i}$ and $\bar{L}$ are expressed in milliseconds.

Because all *N* requests are launched concurrently, the measured request durations include in-batch queueing. Mean request latency is therefore interpreted as the mean completion time within a concurrent batch of size *N*, rather than as a single-user response time. Both validation modes were measured under identical offered-load conditions.

This request-to-response timing is conceptually consistent with Hyperledger’s definition of read latency. However, the system under test in that reference excludes client APIs and external caches, whereas the present evaluation includes the Gateway and its local Cache [43]. The request latency measurement ends upon receipt of the complete response, consistent with JMeter’s elapsed-time definition [42].

*Throughput* refers to the number of successfully processed requests completed per second. This definition is consistent with the request-rate calculation documented by ApacheBench and JMeter [41,42]. Throughput was calculated as shown in Equation (3).

(3) $$\mathrm{Throughput}\;\left(\mathrm{req}/s\right)=\frac{\mathrm{Number}\;\mathrm{of}\;\mathrm{Successful}\;\mathrm{Requests}}{\mathrm{BCT}\;(s)}$$

All three metrics were calculated separately for each run, and the reported values represent the mean ± sample standard deviation across the five repetitions.

#### 4.3.1. Ledger Validation Results

Table 7 presents the Ledger validation results.

**Table 7:** Ledger validation performance (mean ± sample standard deviation (SD), n=5).

Concurrent Requests	Batch Completion Time (s)	Mean Request Latency (ms)	Throughput (req/s)
50	0.25±0.06	116.28±34.62	212.45±42.75
100	0.42±0.04	202.34±18.73	241.02±20.18
200	0.79±0.03	375.63±12.00	253.02±10.72
500	2.12±0.21	967.30±109.54	238.08±23.09
1000	4.52±0.19	2099.45±100.92	221.59±9.11

*Note:* Batch Completion Time = batch wall-clock time (s); Mean Request Latency = arithmetic mean of the individually measured request durations in each run (ms); Throughput = the number of successfully processed valid access requests per second (req/s).

The mean batch completion time increased from 0.25±0.06 s at 50 concurrent requests to 4.52±0.19 s at 1000 requests. Mean request latency ranged from 116.28 ms to 2099.45 ms, while mean throughput ranged from 212.45 req/s to 253.02 req/s. At every request count and repetition, the number of Ledger queries equaled the request count, while cache hits and cache size remained zero. These counters confirm that every validation request in this mode retrieved the authoritative state from the Ledger.

#### 4.3.2. Cache Validation Results

Table 8 presents the steady-state Cache validation results.

**Table 8:** Cache validation performance (mean ± SD, n=5).

Concurrent Requests	Batch Completion Time (s)	Mean Request Latency (ms)	Throughput (req/s)
50	0.13±0.01	44.38±6.10	397.22±16.96
100	0.27±0.01	98.13±2.71	368.32±15.60
200	0.52±0.01	201.88±17.12	382.78±9.30
500	1.40±0.08	560.61±50.51	358.19±20.28
1000	2.80±0.14	1120.88±56.49	358.16±18.18

*Note:* Batch Completion Time = batch wall-clock time (s); Mean Request Latency = arithmetic mean of the individually measured request durations in each run (ms); Throughput = the number of successfully processed valid access requests per second (req/s).

The mean batch completion time increased from 0.13±0.01 s at 50 concurrent requests to 2.80±0.14 s at 1000 requests. Mean request latency ranged from 44.38 ms to 1120.88 ms, while mean throughput ranged from 358.16 req/s to 397.22 req/s. At every request count and repetition, the number of cache hits equaled the request count, no Ledger queries occurred during the timed interval, and the cache contained one authoritative token-state entry. These counters confirm that the reported measurements represent steady-state cache hits rather than per-request Ledger retrieval.

#### 4.3.3. Comparative Results

Table 9 directly compares the two validation modes.

**Table 9:** Ledger versus Cache performance (mean ± SD, n=5).

Concurrent Requests	Batch Completion Time (BCT) (s)		Mean Request Latency (ms)		Throughput (req/s)	
	Ledger	Cache	Ledger	Cache	Ledger	Cache
50	0.25±0.06	0.13±0.01	116.28±34.62	44.38±6.10	212.45±42.75	397.22±16.96
100	0.42±0.04	0.27±0.01	202.34±18.73	98.13±2.71	241.02±20.18	368.32±15.60
200	0.79±0.03	0.52±0.01	375.63±12.00	201.88±17.12	253.02±10.72	382.78±9.30
500	2.12±0.21	1.40±0.08	967.30±109.54	560.61±50.51	238.08±23.09	358.19±20.28
1000	4.52±0.19	2.80±0.14	2099.45±100.92	1120.88±56.49	221.59±9.11	358.16±18.18

*Note:* Both modes verify the Ed25519 signature and compare the signed claims with the authoritative token state. They differ only in whether that state is retrieved from the Ledger or the synchronized cache during the timed validation interval.

**Figure 5:**
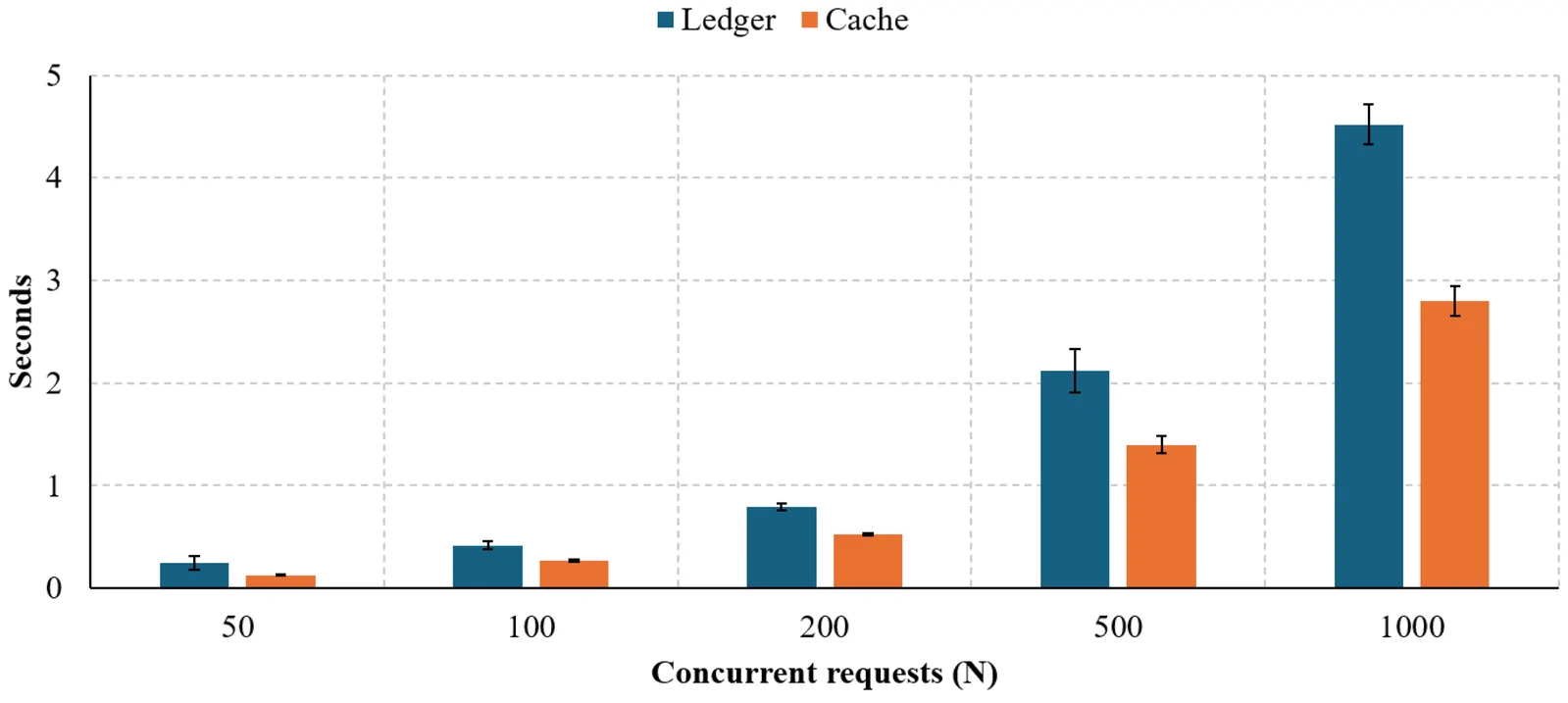
Batch Completion Time comparison between Ledger and Cache validation. Error bars represent sample SD across five runs (n=5).

**Figure 6:**
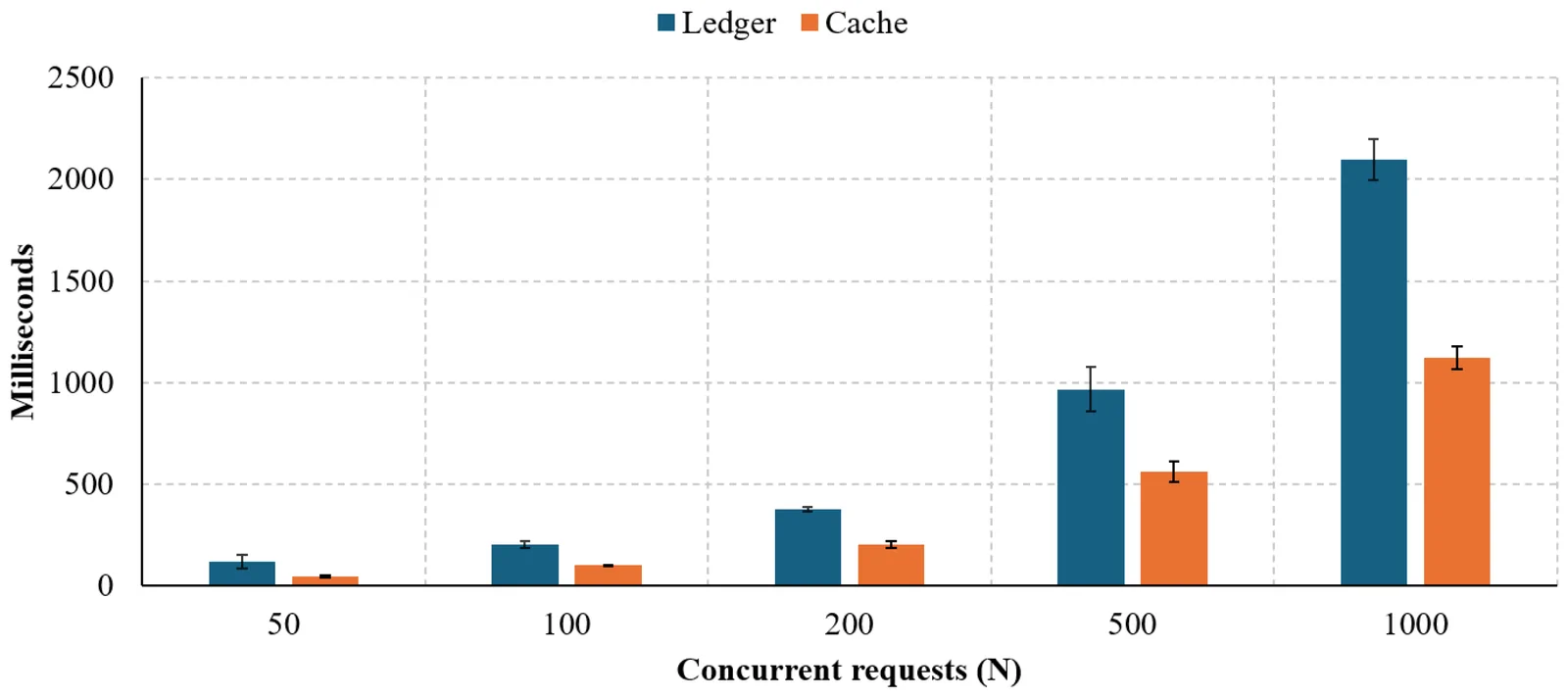
Mean Request Latency comparison between Ledger and Cache validation. Error bars represent sample SD across five runs (n=5).

**Figure 7:**
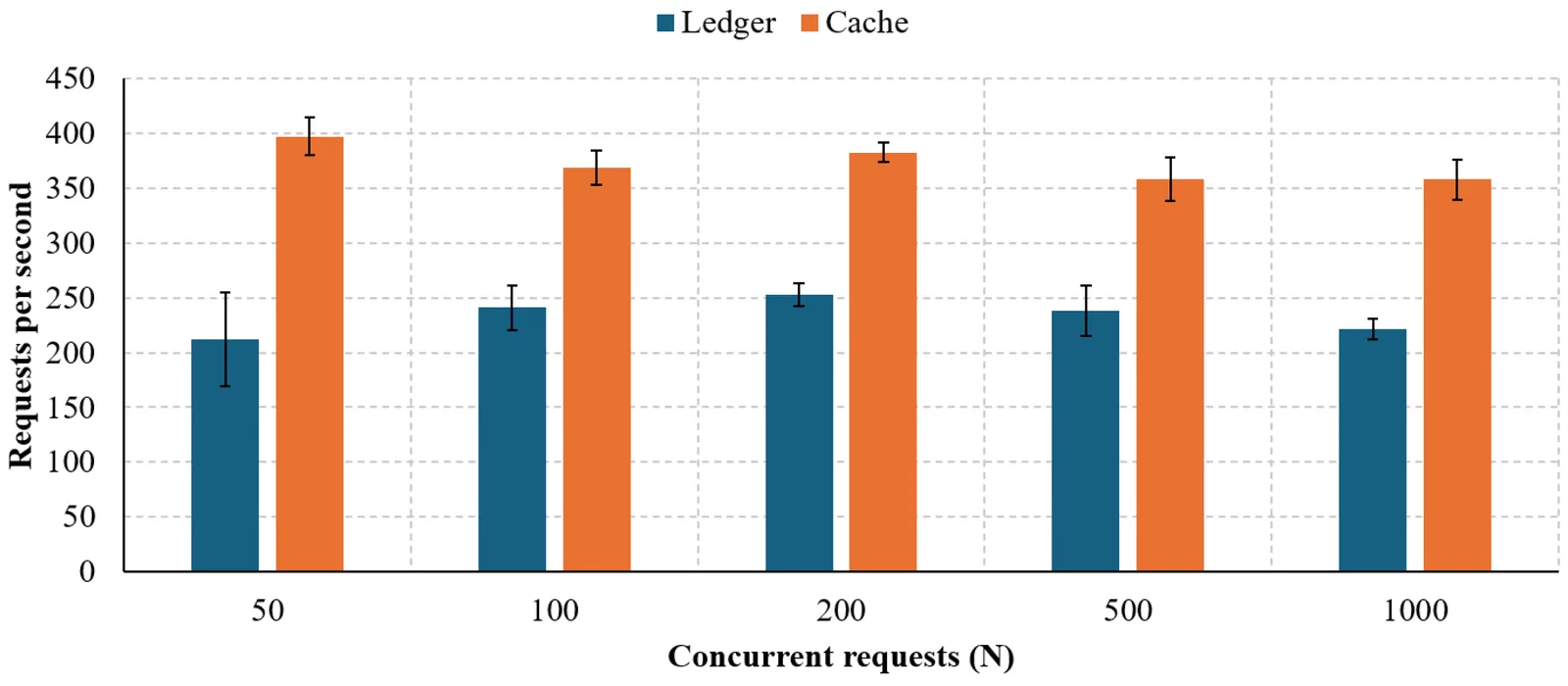
Throughput comparison between Ledger and Cache validation. Error bars represent sample SD across five runs (n=5).

Cache validation consistently outperformed Ledger validation at every tested request count. Across the five loads, the cache reduced mean request latency by approximately 42.04–61.83% and achieved approximately 1.50–1.87 times the throughput of Ledger validation. At 1000 concurrent requests, Cache mode reduced mean batch completion time from 4.52±0.19 s to 2.80±0.14 s. Mean request latency decreased from 2099.45±100.92 ms to 1120.88±56.49 ms, corresponding to an approximately 46.61% reduction. Mean throughput increased from 221.59±9.11 req/s to 358.16±18.18 req/s, representing approximately 1.62 times the Ledger-mode throughput. Because both modes performed the same signature verification and signed-claim comparison, the comparison captures the performance difference between Ledger queries through a persistent Fabric Gateway SDK connection and validation using the synchronized cache. Figure 5, Figure 6 and Figure 7 illustrate the comparison between the two validation modes.

Figure 5 shows that mean batch completion time increased with the request count in both modes. However, Cache validation consistently required less time than Ledger validation, with the difference becoming more pronounced at higher loads.

Figure 6 shows that Cache validation maintained substantially lower mean request latency than Ledger validation at every tested request count.

Figure 7 shows that Cache validation consistently achieved higher throughput than Ledger validation. Under the evaluated workload and in the steady-state Cache condition, validation using synchronized Ledger-derived token state was more efficient than performing a Ledger query through the persistent Fabric Gateway SDK connection for every request.

#### 4.3.4. Multi-User and Multi-Token Validation

The experimental setup is described in Section 4.1. All measured requests completed successfully.

Table 10 reports the mean and sample standard deviation across the five repetitions. At 1000 concurrent requests, Cache mode reduced mean request latency from 2003.52 ms to 1014.65 ms and batch completion time from 4.48 s to 2.70 s. Mean throughput increased from 223.83 to 370.01 requests per second, corresponding to 1.65 times the Ledger-mode throughput. The result shows that the performance difference between the two validation modes persisted when requests were distributed across multiple identities and tokens under the evaluated conditions.

#### 4.3.5. Effect of Cache Hit Ratio

The experimental setup is described in Section 4.1. All 10,000 measured requests completed successfully, and the observed hit ratios matched the planned ratios.

As shown in Table 11, mean request latency decreased as the cache-hit ratio increased, while throughput increased. Between the 0% and 100% conditions, mean request latency decreased from 232.82 ms to 88.34 ms, a reduction of 62.06%, and mean throughput increased from 169.21 to 329.52 requests per second, corresponding to 1.95 times the 0% value. Mean batch completion time decreased from 0.613 s to 0.305 s. These results show that the measured benefit of cache-assisted validation depends on the proportion of requests served from the local cache under the evaluated conditions.

#### 4.3.6. Effect of Cache Capacity

The experimental setup is described in Section 4.1. All 100,000 measured requests completed successfully. In every run, the number of Ledger queries equaled the number of cache misses. As shown in Table 12, capacities below the 100-token working set produced repeated cache misses and LRU evictions. Increasing capacity reduced mean request latency and increased throughput.

When capacity increased from 25 to 100 records, the observed cache-hit ratio increased from 23.09% to 100%, mean request latency decreased from 222.24 ms to 111.02 ms, and throughput increased from 294.32 to 582.55 requests per second. Mean misses per run decreased from 769.05 to zero, while mean evictions decreased from 751.95 to zero. At a capacity of 100 records, the complete working set fit in the cache, and latency and throughput were close to those of the unbounded reference.

These absolute values should not be compared directly with those in Table 11, because the cache-capacity experiment used 1000 requests per run with a client concurrency of 64, whereas the cache-hit-ratio experiment used batches of 100 concurrent requests.

#### 4.3.7. Effect of Emulated Network Delay

The effect of network delay was evaluated by applying tc/netem to the communication path between the persistent Fabric Gateway SDK service and peer0.org1.example.com. Added round-trip times of 0, 50, 100, and 200 ms were emulated by applying half of the target delay in each direction to traffic on port 7051. Client-to-Gateway traffic was not delayed. The experiment used 100 signed tokens issued to ten Fabric identities, batches of 100 requests issued with a concurrency of 64, and 20 repetitions for each validation mode and delay level. All 16,000 measured requests completed successfully. In Cache mode, all timed requests were served as cache hits and no Fabric SDK queries were issued.

Table 13 shows that Ledger-mode validation was increasingly affected by the added Gateway-to-peer delay, whereas Cache-mode validation remained comparatively stable because cache hits did not require a Ledger query. From 0 to 200 ms of added round-trip time, mean Ledger-mode request latency increased from 90.90 ms to 304.22 ms, while throughput decreased from 304.93 to 147.83 requests per second. Over the same interval, mean Cache-mode request latency changed from 16.58 ms to 17.43 ms, and throughput changed from 783.27 to 759.95 requests per second. At 200 ms, Cache mode recorded 94.27% lower mean request latency and 5.14 times the throughput of Ledger mode within this experiment. These measurements represent single-host network emulation and should not be interpreted as results from a geographically distributed deployment.

#### 4.3.8. Token Issuance

The experimental setup is described in Section 4.1. All 100 access requests returned ALLOW, and for each token the Signer returned an Ed25519-signed payload matching the issued token state.

As shown in Table 14, mean Fabric issuance time was 2202.66 ms, whereas the mean Signer-request time was 2.90 ms. Mean end-to-end issuance time was 2206.94 ms. Under the evaluated configuration, most of the measured end-to-end interval lay in the Fabric issuance measurement rather than the Signer request. The event-driven synchronization that follows commit is measured separately in Section 4.3.9 and is not included in the issuance intervals reported here.

#### 4.3.9. TokenIssued Synchronization

The experimental setup is described in Section 4.1. Synchronization was recorded successfully for all 100 tokens.

Table 15 reports the three intervals measured for the synchronization path. The mean interval from event receipt to cache readiness was 22.03 ms, and the mean interval from CLI-observed commit confirmation to cache readiness was 22.52 ms. The corresponding median values were 18.38 ms and 18.97 ms. The largest event-to-cache interval (218.38 ms) occurred for the first measured token; this observation was retained in all summary statistics. The CLI confirmation timestamp and the event-receipt timestamp were captured by independent processes with millisecond resolution. Consequently, one observation placed event receipt 1 ms before CLI confirmation. This observation was retained, and the first interval should be interpreted as the ordering of these externally observed timestamps rather than as an internal peer commit-to-event latency.

#### 4.3.10. Revocation Propagation

Revocation propagation was evaluated under three event-delivery conditions: normal delivery, delayed delivery, and interrupted delivery. Each condition was repeated five times using a newly issued token whose state had been loaded into the Gateway cache before the timed trial. During each trial, the signed token was presented to the Gateway at approximately 10 ms intervals while RevokeToken was invoked. Under normal delivery, the Listener forwarded the committed TokenRevoked event without an intentional delay. Under delayed delivery, a 1000 ms delay was introduced between event receipt and forwarding to the Gateway. Under interrupted delivery, the Listener was stopped before RevokeToken was invoked and was restarted 1000 ms after the invocation returned; it then resumed event delivery from its saved checkpoint. One preliminary execution, used only to synchronize the Listener and its saved checkpoint, was excluded from all reported statistics. All 15 measured trials completed successfully.

Table 16 summarizes the observed revocation intervals. Under normal delivery, the cached status was updated an average of 9.15 ms after the RevokeToken invocation returned, and the first rejected request was observed after 16.79 ms. With the 1000 ms forwarding delay, these intervals increased to 1011.29 ms and 1017.72 ms, respectively. When event delivery was interrupted, cache readiness occurred after 1415.20 ms on average, and the first rejected request was observed after 1420.29 ms. The interrupted-delivery interval includes the interruption, Listener reconnection, checkpoint-based event replay, and Gateway processing. These results show that Cache-mode revocation is asynchronous: an otherwise valid token may continue to be accepted until the TokenRevoked event has been applied to the cached state.

## 5. Discussion

In the main repeated-validation benchmark, Cache mode achieved lower batch completion time and mean request latency and higher throughput than Ledger mode at every tested concurrency level.

To contextualize the architectural contribution of the proposed framework, Table 17 presents a design-level comparison of the proposed framework with Fabric-IoT [15], BloAC [16], DGAC-IoT [17], and G-CapBAC [20], based on the design characteristics and evidence reported in the respective studies.

The comparison illustrates how the selected frameworks distribute authorization evaluation, state management, caching, and revocation differently. Fabric-IoT and BloAC evaluate ABAC policies on-chain and record operational authorization state on the ledger, while neither study explicitly reports a gateway authorization cache [15,16]. In contrast, DGAC-IoT performs ABAC evaluation off-chain and maintains a validity-bounded authorization-state cache at domain gateways, while anchoring compact signed authorization states, policy commitments, and revocation indicators on-chain; plaintext ABAC policies and attributes remain off-chain [17]. G-CapBAC uses group capability tokens, ring signatures, and blockchain-managed group-capability information, but likewise does not explicitly report such a cache [20]. Access withdrawal also differs: Fabric-IoT [15] updates underlying permissions, and BloAC [16] provides time-bounded access together with user- and device-deletion operations, whereas DGAC-IoT [17], G-CapBAC [20], and the proposed framework provide mechanisms for revoking a previously issued authorization. For the proposed framework, the main architectural difference lies in where the initial authorization decision is evaluated and how the resulting authorization is reused: the initial decision and token-state creation occur in chaincode, and issuance depends on transaction commit, whereas subsequent token validation does not repeat the ABAC evaluation; cache hits additionally avoid Ledger retrieval. This comparison identifies a difference in authorization workflow; it does not establish performance superiority over the compared frameworks.

In the main repeated-validation benchmark, the advantage of Cache mode reflects local token-state retrieval during steady-state validation. This benchmark does not represent end-to-end authorization performance. In addition, the Cache was pre-warmed before measurement, so this benchmark does not include cold-start or cache-population costs.

As noted in Section 4.1, these concurrency results apply to a single Smart Gateway instance and do not demonstrate horizontal scale-out across multiple Gateway instances.

The additional experiments show that the performance benefit of Cache-mode validation is not fixed. It remained observable with multiple users and tokens, but increased as the cache-hit ratio increased. The benefit therefore depends on workload locality and on the availability of the required token state in the cache. The cache-capacity experiment showed that, for a fixed working set, capacities below the number of active tokens led to repeated cache misses and evictions. Smaller capacities were associated with higher mean request latency and lower throughput. This result was obtained using an experimental entry-count LRU cache, a working set of 100 tokens, and equal request frequencies across tokens. It does not establish performance under memory-based limits, other eviction policies, or skewed access patterns.

The network-delay results indicate that repeated Ledger-mode validation is sensitive to delay on the Gateway-to-peer path, whereas cache hits reduce this dependence by avoiding a peer query. The issuance measurements also show that the initial authorization path is dominated by the Fabric issuance stage rather than by the Signer request. In contrast, the revocation experiments demonstrate that asynchronous cache updates introduce a freshness trade-off: the interval during which a revoked token whose cached status is still Active may be accepted increases when event forwarding is delayed or the Listener is interrupted. Checkpoint-based replay restored event processing after the tested interruption, but the results do not establish immediate consistency between the Ledger and the Cache.

The functional tests show that the evaluated enforcement path rejects modified, expired, revoked, and device- or action-mismatched tokens, while ABAC rejects requests that do not satisfy the applicable policy. These results support the integrity, validity, revocation, and device–action binding properties exercised by the test cases; they should not be interpreted as a complete security analysis beyond those cases. The access-time condition extends the policy beyond role, department, device, and action, but it was validated functionally rather than under performance workloads.

## 6. Conclusions

The framework was implemented as a proof-of-concept and evaluated in both Ledger mode, using a persistent Fabric Gateway SDK connection, and Cache mode. The experimental results demonstrated that Cache mode reduced mean batch completion time and mean request latency while increasing mean throughput compared with Ledger-based validation. At 1000 concurrent requests, Cache-based validation reduced mean request latency, calculated from individually measured request durations, from 2099.45 ms to 1120.88 ms, a reduction of 46.61%, and increased mean throughput from 221.59 req/s to 358.16 req/s, corresponding to 1.62 times the Ledger-mode throughput. These findings indicate that Cache-assisted validation can effectively reduce repeated Ledger-query overhead while preserving secure access-validation checks. Overall, under the evaluated proof-of-concept conditions, the implemented access-control functions operated as intended. The results do not, however, demonstrate production readiness or horizontal scalability.

The current prototype has several limitations. First, the off-chain Signer is part of the trusted computing base. In the prototype, the Signer loads its Ed25519 private key from a local, unencrypted PEM file, and the Smart Gateway holds only the corresponding public key for signature verification. Production-grade key storage, key rotation, and compromise-recovery procedures are not implemented or evaluated. Validation against the authoritative token state provides an additional check on the signed claims, but it does not eliminate the risk of Signer-key compromise; in Cache mode, this check also depends on the freshness of the cached token state. The key-management measures discussed in this paper, including Key Management Service-based protection, threshold signing, key rotation, and compromise recovery, are proposed safeguards for future deployment. They are not implemented or experimentally validated in the current prototype. Future work will examine their implementation and integration with the Gateway’s signature-verification process.

Second, the proof-of-concept evaluation used simulated IoT devices and a single Smart Gateway, with all components deployed on one host under Windows Subsystem for Linux 2 (WSL2) and one peer configured for each organization. This controlled environment supported functional validation and a comparison of Ledger and Cache modes under the same conditions, but it does not represent a geographically distributed deployment or horizontal scaling across multiple Gateway instances. Running all components on one host may influence the measurements in two ways. Local communication avoids the physical network paths of a distributed deployment, while competition for shared central processing unit (CPU), memory, and networking resources may affect request scheduling and processing times. Both factors may influence the measured latency and throughput, including the observed difference between Ledger and Cache modes. Their individual effects cannot be quantified from the present experiments. The network-delay experiment examined added delay on the Gateway-to-peer path under otherwise unchanged conditions. It did not reproduce the variable delay, packet loss, bandwidth constraints, or independently provisioned hosts of a real wide-area deployment. These results therefore describe sensitivity to the configured delay within the experimental environment and should not be interpreted as measured wide-area network performance. Future work will evaluate the framework across multiple hosts, peers, and Gateway instances and with physical IoT devices under more realistic network conditions.

Third, the main Ledger-versus-Cache benchmark represents steady-state repeated signed-token validation after cache warm-up and an untimed preflight. Token issuance and TokenIssued synchronization were measured separately rather than as part of the timed validation interval. Accordingly, the benchmark results characterize repeated token validation and should not be interpreted as a combined end-to-end measurement of the initial authorization, issuance, synchronization, and subsequent validation stages.

Fourth, the comparison with Fabric-IoT [15], BloAC [16], DGAC-IoT [17], and G-CapBAC [20] was conducted at the design level rather than as a direct performance benchmark. These studies used different experimental environments, workloads, configurations, and evaluation metrics. Therefore, the comparison should be interpreted as a qualitative design-level comparison rather than a direct measurement under identical conditions. Future work will consider re-implementing selected related frameworks in the same experimental environment to enable a fairer and more controlled performance comparison.

Fifth, the prototype cache used in the main experiments has no configured capacity limit. Bounded capacity was evaluated separately using an experimental entry-count LRU extension, a fixed working set of 100 tokens, and equal request frequencies across tokens. Memory-based limits, other eviction policies, and skewed access patterns were not evaluated. Attribute heterogeneity across identities was not evaluated. In addition, although checkpoint-based replay after a Listener interruption was exercised, the Gateway non-acknowledgment path involving an HTTP 503 response and subsequent event redelivery was not tested separately. The handling of old or duplicate events was likewise not evaluated. The access-time condition relies on the Fabric transaction timestamp supplied in the transaction proposal, and the prototype does not perform an independent clock-skew check. Future work will examine additional cache policies and access patterns, heterogeneous attribute distributions, these event-redelivery cases, and independent time-validation mechanisms.

Finally, the functional validation used a limited set of representative roles and devices, such as doctor, nurse, and patient-monitoring devices. These cases exercised the implemented access-control logic but do not represent a complete healthcare deployment scenario. Future work will extend the evaluation to include more detailed healthcare scenarios, additional user roles, diverse IoT medical devices, emergency access conditions, and broader policy variations. In addition, future work will extend the proposed framework to support secure capability-token delegation, including delegation policies, delegation-depth control, and efficient revocation of delegated access rights in IoT environments.

## Figures and Tables

**Table 1:** Summary of Studies, Access Control Models, and Blockchain Platforms.

Study	Access Control (AC)	Blockchain Platform
[8]	RBAC	Hyperledger Fabric
[9]	ABAC	Hyperledger Fabric
[10]	ABAC	Hyperledger Fabric
[11]	ABAC	Hyperledger Fabric
[12]	ABAC	Ethereum
[13]	ABAC	Hyperledger Fabric
[14]	ABAC	Blockchain, unspecified platform
[15]	ABAC	Hyperledger Fabric
[16]	ABAC	Hyperledger Fabric
[17]	ABAC	Hyperledger Fabric
[18]	CapBAC	Ethereum
[19]	CapBAC	Ethereum
[20]	CapBAC	Hyperledger Fabric
[21]	Hybrid ABAC–RBAC	Ethereum

*Note:* Attribute-Based Access Control (ABAC); Capability-Based Access Control (CapBAC); Role-Based Access Control (RBAC).

**Table 2:** Key Findings and Limitations.

Study|Year	Key Findings	Limitations
[8]|(2024)	Scalable, decentralized, tamper-resistant access control with real-time conflict resolution.	Although the framework addresses policy conflicts through delegation and revocation in the tested scenarios, the authors acknowledge remaining RBAC limitations related to role explosion and manual role assignment; larger networks may expose additional policy conflicts.
[9]|(2022)	Improved throughput, fine-grained access control, and transparent decision-making.	Experiments were conducted on a single-host virtual machine rather than a geographically distributed Fabric deployment; specific access-control attacks such as forgery or man-in-the-middle attacks were not evaluated.
[10]|(2022)	Decentralized and dynamic access control via smart contracts, scalable for IIoT.	Evaluation is restricted to simulation settings; no validation using real industrial gateways or physical IIoT devices.
[11]|(2021)	Low-power IoT integration with ARM64, efficient ABAC execution, and decentralized access control.	Provides only basic performance measurements; lacks extensive testing across different hardware configurations and does not fully assess power or memory constraints.
[12]|(2021)	Scalability and fine-grained access control with multichain architecture and decentralized security.	Relies on complex blockchain and smart-contract operations; scalability and gas cost remain concerns for large-scale IoT deployment.
[13]|(2020)	Emergency access mechanism and decentralized access control for resource-constrained IoT environments using Hyperledger Fabric chaincode.	Co-decision time increases as collaborative nodes grow; experiments were limited to a small-scale setup using three Raspberry Pi devices.
[14]|(2024)	Blockchain and ABE ensure secure and traceable data access and support large attribute universes.	CP-ABE operations introduce computational overhead due to bilinear pairing and LSSS structures, which may limit suitability for constrained IoT devices.
[15]|(2020)	Automated policy enforcement, scalability, decentralization, and reduced administrative overhead.	Experimental validation used only a small setup of two PCs; no testing on edge clusters or diverse physical IoT devices.
[16]|(2024)	Decentralized ABAC using Hyperledger Fabric hosted by edge and gateway nodes. The framework was evaluated through simulation and a four-Raspberry-Pi testbed, with the reported results indicating lower latency than cloud-server-based access control.	The initial setup requires specialized knowledge of blockchain, Hyperledger Fabric, smart contracts, and ABAC policies. The authors also identified scalability and interoperability challenges in large-scale deployments and noted that the static assignment of blockchain roles to edge nodes may create a performance bottleneck.
[17]|(2026)	Off-chain gateway-based ABAC with validity-bounded authorization caching and Hyperledger Fabric-based trust anchoring. The framework was evaluated under increasing access-request rates, different cache-hit ratios, and dynamic revocation conditions, with the reported results indicating reduced access latency and improved throughput.	The evaluation was limited to a controlled prototype environment using emulated IoT devices and generated access-control workloads rather than a large-scale physical IoT field deployment.
[18]|(2020)	Action-level capability tokens and graph-based delegation implemented using Ethereum smart contracts. Gas consumption was evaluated for token creation, delegation, and revocation.	Unencrypted on-chain tokens limit access-right privacy. Real-world IoT deployment and validation of security goals remain future work.
[19]|(2021)	Decentralized identity management, feasible and granular access control, and tamper-proof logs.	Capability credential verification may cause high latency on constrained edge devices; scalability depends on blockchain parameter tuning such as block size and block interval.
[20]|(2025)	G-CapBAC improves CapBAC storage efficiency and authentication/access speed using group capability tokens and ring signatures.	Performance is sensitive to group size and access-pattern assumptions; evaluation is simulation-based and does not include physical IoT energy-consumption measurements.
[21]|(2023)	Hybrid ABAC–RBAC for secure and dynamic access control, scalable for smart farming environments.	Evaluation does not report transaction throughput or network latency; active attacks such as DDoS or man-in-the-middle attacks were not experimentally tested.

*Note:* Attribute-Based Encryption (ABE); Ciphertext-Policy Attribute-Based Encryption (CP-ABE); Distributed Denial-of-Service (DDoS); Industrial Internet of Things (IIoT); Linear Secret-Sharing Scheme (LSSS); personal computers (PCs).

**Table 4:** On-chain and off-chain design decisions.

Component	Placement	Justification
ABAC policies	On-chain	The Hybrid ABAC–CapBAC Chaincode stores the policies used to evaluate role, department, device, and action attributes. On-chain storage provides a shared, auditable, and tamper-resistant policy source.
X.509 user attributes	Fabric identity layer; evaluated by chaincode	The chaincode reads the role and department attributes from the MSP-validated X.509 client identity using the GetAttributeValue() method provided by Fabric’s ClientIdentity interface [35]. The MSP validates the client certificate and its issuing certification authority [31]. These attributes are not stored as separate identity records, although selected values are copied into the on-chain token state.
Capability-token state	On-chain	The ledger stores tokenId, tokenRef, subject, MSP ID, role, department, device ID, action, expiry, and status. This state provides an auditable authorization record and remains authoritative for validation and revocation.
Token status (revocation)	On-chain	An administrator invokes RevokeToken(tokenId) to change the token status to Revoked. After commit, a TokenRevoked event is forwarded to the Gateway, which updates the cached token status and removes the corresponding cached signed token.
Fabric transaction pipeline	Fabric network	Endorsing peers execute and endorse the chaincode according to the AND(’Org1MSP.peer’,’Org2MSP.peer’) policy. The transaction is then ordered using Raft, validated by the peers, and committed to the Ledger.
Full signed capability token	Off-chain	The Signer returns the signed token to the Gateway. The Gateway verifies and caches it, after which the user retrieves it using tokenId. The complete signed token is not stored on the Ledger.
Token signing	Off-chain	A separate Signer service signs selected authorization claims using an Ed25519 private key. Keeping the private key outside the chaincode avoids embedding confidential signing material in the chaincode or storing it on the Ledger.
Event Listener	Off-chain	The Listener receives committed TokenIssued and TokenRevoked events and forwards their event data to the Gateway API. The Gateway then retrieves or updates the corresponding authoritative token state.
Gateway authorization cache	Off-chain	The in-memory cache stores synchronized token states and verified signed tokens. Committed events synchronize its state, while a Ledger query is used on a cache miss. Reusing cached token state reduces repeated Ledger queries during Cache-mode validation.
Signature verification	Off-chain	Before authoritative state validation, the Gateway verifies the Ed25519 signature using the Signer’s pre-provisioned public key. The current token state is then obtained through either a direct Ledger query or the event-driven cache.
IoT interactions	Off-chain; simulated	The Gateway returns a simulated IoT action result after successful validation. The current prototype does not forward commands to or actuate physical IoT devices.
Users and client applications	Off-chain	Users and administrators interact through Fabric clients and Gateway REST endpoints rather than operating as blockchain nodes. Users request authorization through Fabric and retrieve and submit signed tokens through the Gateway API.

*Note:* Identifier (ID); Representational State Transfer (REST).

**Table 10:** Multi-user and multi-token validation results. Values are mean ± sample standard deviation across five repetitions.

Concurrent Requests	Mode	Batch Completion Time (s)	Mean Request Latency (ms)	Throughput (Requests/s)
50	Ledger	0.507±0.652	110.09±21.45	192.52±92.71
50	Cache	0.133±0.009	42.95±2.77	377.93±23.88
100	Ledger	0.456±0.059	225.68±45.06	222.08±27.46
100	Cache	0.243±0.005	85.28±5.17	412.28±9.09
200	Ledger	0.857±0.074	414.63±45.04	234.89±20.20
200	Cache	0.531±0.057	195.34±22.97	379.95±36.64
500	Ledger	2.222±0.122	993.32±42.31	225.58±12.76
500	Cache	1.346±0.088	502.31±44.25	372.66±24.34
1000	Ledger	4.481±0.265	2003.52±115.28	223.83±14.14
1000	Cache	2.704±0.079	1014.65±42.88	370.01±10.73

All requests were distributed across ten Fabric identities and up to 100 distinct signed tokens. Cache preparation was performed outside the timed interval.

**Table 11:** Results at different planned cache-hit ratios. Values are mean ± sample standard deviation across 20 repetitions.

Cache-Hit Ratio	Batch Completion Time (s)	Mean Request Latency (ms)	Throughput (Requests/s)
0%	0.613±0.134	232.82±52.30	169.21±29.48
25%	0.528±0.066	186.39±29.02	192.24±22.90
50%	0.437±0.036	148.77±15.70	230.02±17.35
75%	0.394±0.089	125.52±34.12	261.31±37.04
100%	0.305±0.022	88.34±8.36	329.52±22.20

Each condition comprised 20 batches of 100 concurrent requests. Cache preparation was performed outside the timed interval.

**Table 12:** Cache-capacity results with a fixed workload of 100 tokens and 1000 requests per run. Values are mean ± sample standard deviation across 20 repetitions.

Capacity (Records)	Observed Hit Ratio (%)	Misses per Run Evictions per Run	Batch Completion Time (s)	Mean Request Latency (ms)	Throughput (Requests/s)
25	23.09±1.21	769.05±12.12 751.95±11.26	3.665±1.174	222.24±72.02	294.32±73.33
50	46.34±1.47	536.55±14.71 523.50±14.13	3.087±0.801	186.23±48.68	343.94±81.27
75	71.78±1.32	282.25±13.24 274.00±12.24	2.638±0.822	157.86±49.25	412.71±113.92
100	100.00±0.00	0.00±0.00 0.00±0.00	1.879±0.624	111.02±37.41	582.55±161.78
Unbounded	100.00±0.00	0.00±0.00 0.00±0.00	1.904±0.664	112.95±39.86	584.16±178.83

Each run comprised 1000 requests for 100 tokens, with ten requests per token and a client concurrency of 64. Cache misses and evictions were measured during the timed interval; cache preparation was excluded. Each cache miss generated one Ledger query through the persistent Fabric Gateway Software Development Kit (SDK) connection.

**Table 13:** Ledger- and Cache-mode results under emulated Gateway-to-peer network delay. Values are mean ± sample standard deviation across 20 repetitions.

Added Round-Trip Time (RTT) (ms)	Mode	Batch Completion Time (s)	Mean Request Latency (ms)	Throughput (Requests/s)
0	Ledger	0.331±0.035	90.90±6.45	304.93±24.22
0	Cache	0.128±0.011	16.58±1.86	783.27±57.68
50	Ledger	0.374±0.034	131.57±10.31	269.36±20.63
50	Cache	0.126±0.005	15.88±0.74	791.73±28.84
100	Ledger	0.386±0.020	157.28±7.26	259.95±13.23
100	Cache	0.131±0.019	17.29±2.57	772.01±78.22
200	Ledger	0.678±0.037	304.22±11.98	147.83±6.66
200	Cache	0.135±0.028	17.43±3.75	759.95±98.76

The added delay affected only the Fabric SDK service–peer communication path. Client–Gateway traffic was not delayed.

**Table 14:** Token-issuance measurements for 100 tokens issued to ten Fabric identities. Timing values are in milliseconds.

Statistic	Fabric Issuance	Signer Request	End-to-End Issuance
Tokens (*n*)	100	100	100
Mean	2202.66	2.90	2206.94
Sample SD	32.89	0.97	33.30
Median	2193.35	2.53	2197.12
Minimum	2164.95	2.30	2168.50
Maximum	2404.09	7.89	2410.40

Fabric issuance was measured from launching the peer command-line interface (CLI) invocation until the CLI reported VALID commit confirmation at both peers. The Signer measurement represents a separate Hypertext Transfer Protocol (HTTP) request to the Signer. Event-driven synchronization is excluded and reported separately.

**Table 15:** TokenIssued synchronization intervals for 100 issued tokens. Timing values are reported in milliseconds.

Measured Interval	*n*	Mean ± Sample SD (ms)	Median (ms)	Min (ms)	Max (ms)
CLI-observed VALID confirmation at peer0.org1 to event receipt	100	0.49±0.80	0.00	−1.00	6.00
Event receipt to Gateway cache readiness	100	22.03±20.50	18.38	15.90	218.38
CLI-observed VALID confirmation at peer0.org1 to Gateway cache readiness	100	22.52±21.06	18.97	15.95	224.38

The CLI timestamp represents the externally observed VALID confirmation for peer0.org1, rather than the peer’s internal commit timestamp. The −1 ms observation reflects the ordering and resolution of timestamps recorded by independent processes and was retained in the summary.

**Table 16:** Revocation propagation under normal, delayed, and interrupted event delivery. Values are mean ± sample standard deviation across five repetitions.

Event-Delivery Condition	Event Receipt to Cache Readiness (ms)	Invocation Return to Cache Readiness (ms)	Invocation Return to First Rejection (ms)	Accepted Requests After Invocation Return
Normal	9.03±0.30	9.15±0.81	16.79±3.27	0.8±0.45
Delayed by 1000 ms	1010.35±0.38	1011.29±0.20	1017.72±3.12	83.8±0.45
Interrupted (restart 1000 ms after return)	9.36±1.98	1415.20±59.63	1420.29±59.32	115.0±4.74

Invocation return denotes the client-observed completion time of RevokeToken, not the peer’s internal commit timestamp. The invocation returned only after the CLI reported VALID commit confirmation at both peers. The mean interval from invocation return to event receipt was 0.12 ms under normal delivery, 0.95 ms under delayed delivery, and 1405.83 ms under interrupted delivery. In the delayed condition, the event-receipt timestamp was recorded before the configured 1000 ms forwarding delay; therefore, the event-to-cache interval includes that delay. In the interrupted condition, event receipt occurred after reconnection and checkpoint-based replay, so the longer interruption is represented by the invocation-return-to-cache interval.

**Table 17:** Design-level comparison with selected related frameworks.

Framework	Access Model	Gateway Authorization Cache	Operational Authorization State On-Chain	Revocation Support	Reported Evidence
Fabric-IoT [15]	ABAC evaluated on-chain using Hyperledger Fabric smart contracts	–	✓	Limited	Evaluated with 50–1000 concurrent clients; reports cost-time and a consensus-speed comparison.
BloAC [16]	ABAC evaluated on-chain through customized Hyperledger Fabric chaincode	–	✓	Limited	Evaluated through simulation and a four-Raspberry-Pi testbed, with the reported results indicating lower latency than cloud-server-based access control.
DGAC-IoT [17]	ABAC evaluated off-chain at domain gateways, with signed authorization states anchored in Hyperledger Fabric	✓	✓ ^†^	✓	Evaluated under increasing access-request rates, different cache-hit ratios, and dynamic revocation conditions; the reported results indicate reduced access latency and improved throughput.
G-CapBAC [20]	Group CapBAC using capability tokens and ring signatures	–	✓	✓	Reports storage reduction from approximately 400 MB to 41 MB or 4 MB, depending on the group size.
Proposed framework	Hybrid ABAC–CapBAC: initial ABAC evaluation in chaincode, with policies and the complete token state stored on the Ledger and reusable signed capability tokens enforced by the Smart Gateway	✓	✓	✓	At 1000 concurrent requests, Cache mode reduced mean request latency by approximately 46.61% and achieved approximately 1.62 times the throughput of Ledger mode.

*Note:* ✓ = explicitly supported or reported; – = not explicitly reported; Limited = access can be withdrawn by modifying underlying permissions or user/device records, but no dedicated mechanism for revoking a previously issued authorization is reported. Gateway authorization cache refers to the local reuse of previously established authorization state. Operational authorization state on-chain indicates whether the policy, permission, group-capability, or token state used to make or validate access decisions is itself recorded on the ledger, rather than only a cryptographic commitment to that state. For DGAC-IoT, † indicates that compact signed authorization states, rather than plaintext ABAC policies or attributes, are recorded on-chain. DGAC-IoT caches validity-bounded authorization states, whereas the proposed framework caches Ledger-derived token state. For Fabric-IoT, revocation refers to updating the permission attribute in the access policy. BloAC reports time-bounded access and user- and device-deletion operations. The comparison is based on the design characteristics and published evidence reported by each study. It is not a direct performance benchmark because the studies used different experimental environments, workloads, configurations, and evaluation metrics.

## Data Availability

The data supporting the findings of this study are included in the Supplementary Materials. Further inquiries can be directed to the corresponding author.

## Supplementary Materials

The following supporting information can be downloaded at: https://www.mdpi.com/article/10.3390/s26217072/s1, File S1: Raw and summary CSV results for the experiments reported in this study, comprising eight dataset groups within the archive: S1, main repeated-validation benchmark; S2, multi-user and multi-token validation; S3, effect of cache-hit ratio; S4, effect of emulated network delay; S5, token issuance; S6, TokenIssued synchronization; S7, revocation propagation; S8, effect of cache capacity.

## Abbreviations

The following abbreviations are used in this manuscript:

ABAC	Attribute-Based Access Control
CapBAC	Capability-Based Access Control
RBAC	Role-Based Access Control
IoT	Internet of Things
MSP	Membership Service Provider
CA	Certificate Authority
API	Application Programming Interface
ABE	Attribute-Based Encryption
CP-ABE	Ciphertext-Policy Attribute-Based Encryption
IIoT	Industrial Internet of Things
DID	Decentralized Identifier
LSSS	Linear Secret-Sharing Scheme
BCT	Batch Completion Time
SDK	Software Development Kit
HTTP	Hypertext Transfer Protocol
JSON	JavaScript Object Notation
REST	Representational State Transfer
SD	Standard Deviation
CPU	Central Processing Unit
RAM	Random Access Memory
LTS	Long-Term Support
DDoS	Distributed Denial-of-Service
gRPC	gRPC Remote Procedure Calls
LRU	Least Recently Used
ID	Identifier
CLI	Command-Line Interface
PEM	Privacy-Enhanced Mail
UTC	Coordinated Universal Time
RTT	Round-Trip Time
WSL2	Windows Subsystem for Linux 2
